# The Evolution of Phytochemical and Medicinal Plant Research in Chile: Status, Opportunities, and Challenges

**DOI:** 10.3390/plants15142135

**Published:** 2026-07-10

**Authors:** Gonzalo Fuentes-Barros, Sebastián Castro-Saavedra, Nicolás Montalva, Alejandro Vega-Muñoz, Jaime Mella, Antonia Díaz-Valdés, Camila Zoppi, Ana Caroline Avanco, Marco Mellado, Javier Echeverría

**Affiliations:** 1 SAPHYCHEM, Santiago 7550000, Chile; gonzalojfb@gmail.com (G.F.-B.); sebastian.castro.sa@usach.cl (S.C.-S.); camizoppi@gmail.com (C.Z.); 2 Programa de Doctorado en Políticas Públicas, Universidad Mayor, Santiago 8340589, Chile; ana.avanco@mayor.cl; 3 Departamento de Ciencias del Ambiente, Facultad de Química y Biología, Universidad de Santiago de Chile, Santiago 9170022, Chile; 4 Centro de Investigación en Sociedad y Salud (CISS), Universidad Mayor, Santiago 8340589, Chile; nicolas.montalva@umayor.cl (N.M.); antonia.diazvaldes@umayor.cl (A.D.-V.); 5 Facultad de Medicina y Ciencias de la Salud, Universidad Central de Chile, Santiago 8330507, Chile; alejandro.vega@ucentral.cl; 6 Centro de Investigación en Medicina de Altura, Universidad Arturo Prat, Santiago 8340232, Chile; 7 Instituto de Química, Facultad de Ciencias, Universidad de Valparaíso, Valparaíso 2360102, Chile; jaime.mella@uv.cl; 8 Centro de Investigación, Desarrollo e Innovación de Productos Bioactivos (CInBIO), Universidad de Valparaíso, Valparaíso 2360102, Chile; 9 Dirección de Investigación, Universidad Bernardo O’Higgins, Santiago 8370993, Chile; 10 Laboratorio de Espectroscopía y Química Aplicada, Grupo de Investigación en Ciencias Biomédicas Aplicadas, Universidad Central de Chile, Santiago 8330546, Chile

**Keywords:** natural products, phytochemistry, bibliometric analysis, medicinal plants, scientific production

## Abstract

Background: The global use of herbal medicines and research on plant bioactive compounds have steadily increased. In Chile, this growth is linked to ethnobotanical knowledge and advances in phytochemistry, although gaps persist in its evolution, leadership, and regulatory articulation. This study analyzes scientific production on phytochemicals and medicinal plants (1976–2025), identifying growth patterns, leading journals, authorship, and collaboration networks. Methodology: A mixed-methods approach combined bibliometric analysis of Web of Science records with inductive qualitative content analysis from researcher interviews. The quantitative phase applied performance analysis and scientific mapping, using indicators such as Price’s, Lotka’s, Bradford’s, and Zipf’s laws to evaluate evolution, productivity, and impact. The qualitative phase explored perceptions of the field’s current state, limitations, and challenges, thereby contextualizing the bibliometric findings. Results and discussion: Analysis of 2149 articles and 6878 authors shows exponential growth (11.46% annually; R^2^ = 0.952), with 674 publications projected by 2035. Productivity is concentrated among key authors and institutions (University of Chile, Concepción, Talca). Research focuses on antioxidant activity and bioactive compounds. Despite progress, gaps persist in clinical validation, technology transfer, and health system integration, while biodiversity and omics are emerging as strategic assets. Conclusions: Phytochemistry in Chile is expanding rapidly, requiring integrated frameworks that combine clinical validation, sustainable biodiversity management, advanced analytical and computational tools, technology transfer, and adaptive public policies to translate scientific knowledge into evidence-based healthcare, biotechnology, and sustainable innovation.

## 1. Introduction

Humans and plant species have maintained a long-standing relationship that has now become an important source of drugs, agrochemicals, cosmetics, and functional foods [1,2]. In recent decades, this interaction has gained new momentum through approaches such as natural products chemistry, biotechnology, metabolomics, and dereplication, as well as conceptual frameworks such as One Health and sustainability, which have significantly expanded the possibilities for identifying and valorizing bioactive compounds [3,4].

Chile, located at the southern extremity of the Americas, harbors biodiversity characterized by high levels of endemism and a longstanding tradition of using plant species, many of which have been passed down intergenerationally by Indigenous communities. Its territorial diversity—spanning from the Atacama Desert to Patagonia, and encompassing Mediterranean sclerophyllous forests, temperate rainforests, and extensive marine ecosystems—constitutes an exceptional setting for the study and development of natural products [5,6,7].

Several species illustrate this therapeutic and biotechnological potential. One example is quillay (*Quillaja saponaria* Mol. [Quillajaceae]), which has been extensively studied for its applications in animal feed models and for its saponins, such as QS-21, which have been used in vaccine adjuvant development [8,9,10,11,12]. Boldo (*Peumus boldus* Mol. [Monimiaceae]) exhibits anti-inflammatory and hepatoprotective properties, supported by both its traditional use and scientific evidence [13,14,15,16,17]. Also included are matico (*Buddleja globosa* Hope [Scrophulariaceae]), bailahuén (*Haplopappus baylahuen* J. Rémy [Asteraceae]), and other species that stand out for their widespread use and experimental validation [13,18,19,20]. Likewise, maqui (*Aristotelia chilensis* (Molina) Stuntz [Elaeocarpaceae]) [21,22,23,24] and murta (*Ugni molinae* Turcz. [Myrtaceae]) [25,26,27,28,29] are also noteworthy, given their extensive traditional use and growing experimental evidence supporting their biological activities, which constitute relevant sources of antioxidant compounds with potential applications in pharmaceutical, nutraceutical, and cosmetic fields.

Although experiences in Chile have demonstrated the potential of research on natural products [30]. Structural gaps persist, limiting its consolidation as a sector with sustained impact on society and the economy. In this regard, strengthening interdisciplinary research, together with the incorporation of the One Health approach and sustainability criteria, is proposed as a strategic pathway toward an integrated valorization of plant biodiversity, with scientific research and development as fundamental pillars. In this context, it is equally important to have analytical tools that enable the characterization and understanding of the field’s development, among which bibliometric techniques stand out.

Nevertheless, although bibliometric techniques are widely used, it is necessary to acknowledge their inherent limitations [31,32,33]. Many descriptive bibliometric studies fail to capture fundamental elements, such as underlying theoretical frameworks, the conceptual quality of contributions, or their practical relevance in specific contexts. This may lead to an oversimplification of a phenomenon that, at its fullest expression, is highly complex and multidimensional [31,32,33,34].

To mitigate these biases, it is essential to complement bibliometric analysis with qualitative approaches, such as the mixed-methods design adopted in this study [34], which enables a deeper and more contextually grounded understanding of the field by exploring the experiences, perceptions, and opinions of the key actors in phytochemical research in Chile [34]. The integration of quantitative and qualitative methods within a mixed-methods design not only enriches the analysis but also strengthens the interpretative validity of the findings [35,36].

Despite this promising scenario, critical questions remain regarding the trajectory of research consolidation in this field, its integrated vision toward sustainability, and its real impact on society [37]. In this context, the present study aims to analyze the evolution of scientific research on plant-derived natural products in Chile over the past fifty years, from a chemical–medicinal exploitation perspective. The investigation was structured around the following research question: What have been the evolutionary dynamics of this field, and what limitations or opportunities for future development define its current state? This analysis seeks to identify the discipline’s strategic contributions across five key dimensions: public health, the productive sector, environmental sustainability, social impact, and strengthening scientific research infrastructure. To address this question, a systematic bibliometric review was conducted, complemented by in-depth interviews with researchers working in phytochemistry and related fields. This approach made it possible to characterize the main research lines, thematic trends, collaboration networks, prevailing methodological approaches, and their articulation with productive and regulatory development.

## 2. Results

### 2.1. Trends and Growth in Publications on Natural Products and Related Topics

**Figure 1:**
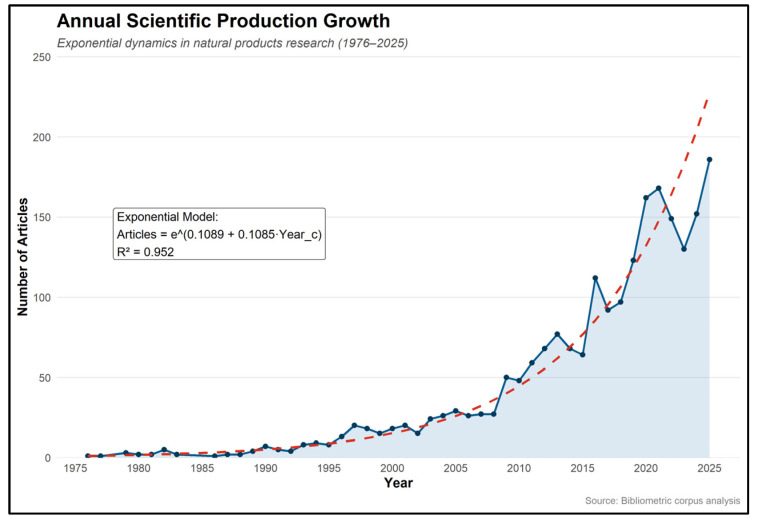
Annual evolution of WoS publications with Chilean affiliation in phytochemistry and natural research (1976–2025). The blue line with circular markers represents the observed annual number of publications, while the red dashed line represents the exponential regression model fitted to the annual publication data. The regression equation and coefficient of determination (R^2^) are shown in the figure.

Between 1976 and 2025, a total of 6878 researchers participated, generating 38,168 records associated with 2149 original articles. In this context, scientific output has shown sustained growth, following a clearly exponential trend. This pattern is supported by a high coefficient of determination (R^2^ = 0.95), indicating a robust model fit (Figure 1). Consistent with this trajectory, the corpus reached an h-index of 86, reflecting a relatively high concentration of highly cited papers within the field (Figure S1).

The model revealed highly significant exponential growth (β = 0.108; *p* < 0.001; R^2^ = 0.952), indicating strong explanatory power and confirming a robust pattern of expansion in the field under study. In its logarithmic form, the model is expressed as follows: $log\left(Articles\right)=0.1089\;+\;0.1085\;Year\text{\_}c$. This coefficient corresponds to an approximate annual growth rate of 11.46%, reflecting a sustained expansion of scientific output. Likewise, the doubling time, estimated using: ln(2)/b, yields approximately 6.4 years, indicating that scientific output doubles roughly every six and a half years (Figure 1). At a sustained growth rate, scientific output is projected to reach approximately 674 articles per year by 2035 (Figure S2).

### 2.2. Prolific Authors and Co-Authorship in the Field of Natural Products and Related Topics

**Figure 2:**
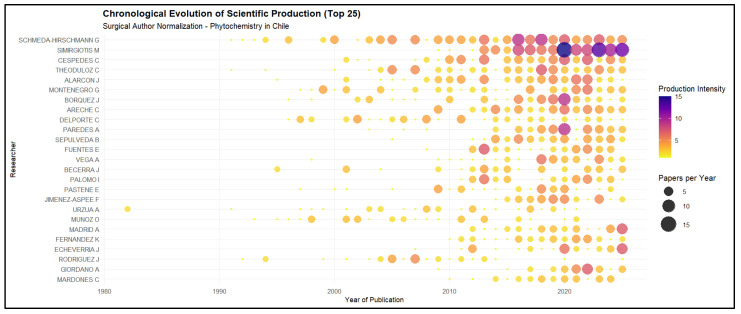
Evolution of publication output among the most prolific scientists over the period 1976–2025.

**Figure 3:**
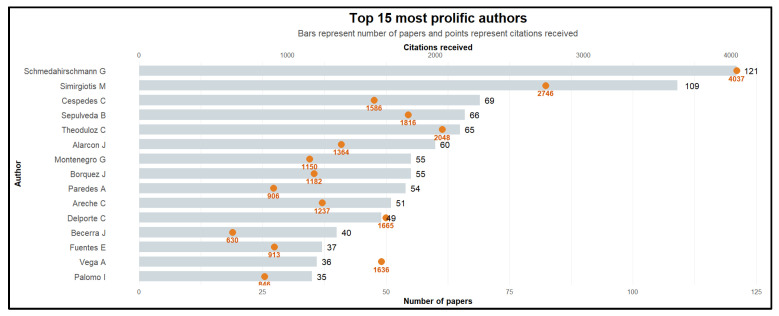
Top 15 Chilean authors in phytochemistry and natural products by publications and citations (1976–2025). Black numbers denote publications, whereas orange numbers denote citations. (For Palomo I, the citation count is 846).

The development of phytochemistry in Chile has involved multiple actors since its inception in the mid-twentieth century, including studies published in local journals that are not indexed in Web of Science (WoS). However, for this study, only records indexed in WoS were included in the analysis, with the oldest article dating back to 1976. In this pioneering stage, Doctors Mario Silva, Bruce Cassels, Glauco Morales, Juan Garbarino, Alejandro Urzúa, and Carla Delporte, among other researchers, played a prominent role in laying the foundations for the field’s development (Figure 2). Today, the field shows a marked concentration of leadership around two central figures: Guillermo Schmeda-Hirschmann (121 publications) and Mario J. Simirgiotis (109 publications), who have become key reference authors in the area (Figure 3). In terms of authorship participation, these researchers have led or co-led nearly 5% of the field’s national scientific output, highlighting a high degree of concentration in knowledge generation.

From a bibliometric standpoint, the citation volume reached by Schmeda-Hirschmann is 4037, and by Simirgiotis is 2746 (Table S1, Supplementary Materials). The volume of publications and citations not only reflects high productivity but also positions Schmeda-Hirschmann’s body of work as the foundational theoretical framework upon which the scientific community builds new knowledge. In particular, his work represents the transition from pioneering researchers to the field’s current leadership. His research has enabled a critical inventory of secondary metabolites and their biological activities and has played a key role in training and dynamizing the national research ecosystem. For his part, the scientific output of Dr. M. Simirgiotis stands out for the high-resolution metabolic profiling of plant species with traditional use and biotechnological potential. His approach integrates advanced analytical chemistry, through liquid chromatography coupled with mass spectrometry, with the search for novel antioxidant, antimicrobial, and neuroprotective agents, all framed within principles of sustainable chemistry.

On the other hand, when analyzing relative impact (citations per document), figures with high scientific efficiency emerge. Dr. Antonio Vega-Gálvez, despite ranking 14th in publication volume, has the highest average impact in the sample (45.5 citations/paper). This indicates that his work exerts exceptional influence and generates substantial international interest. In a similar vein, Dr. Carla Delporte surpasses even the most prolific leaders in relative impact, confirming the quality and relevance of her research in the fields of pharmacognosy and phytochemistry (33.98 citations/paper) (Table S1, Supplementary Materials). Finally, key actors who reinforce the robustness of research groups in phytochemical analysis complete the ranking. Dr. Jorge Bórquez (55 documents; 21.49 citations/paper) and Dr. Carlos Areche (51 publications; 24.25 citations/paper) stand out in this regard.

When Lotka’s law is applied to the corpus of phytochemical research in Chile (1976–2025), an exponent *k* = 1.93 is obtained, close to the classical theoretical value described by Lotka (*k* = 2). The distribution of author productivity showed a high degree of fit (R^2^ = 0.913), indicating a strongly concentrated structure in which a small group of highly prolific authors contributes disproportionately to the total scientific output. In contrast, 72.2% of researchers recorded only a single publication, reflecting a broad base of occasional participation. This pattern reveals a hierarchical organization of scientific activity and highlights the central role of consolidated research cores in the field’s continuity, specialization, and development (Figure S3).

**Figure 4:**
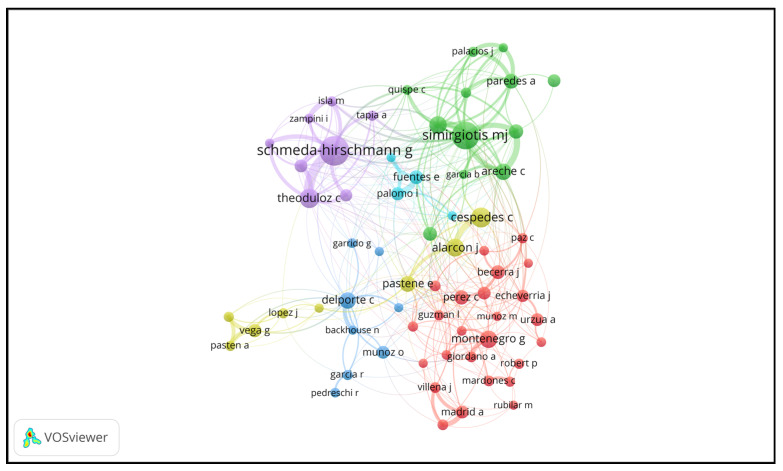
Co-authorship network of the most prolific researchers in phytochemistry (1976–2025). The visualization employs a co-authorship unit analysis based on the association strength normalization method. Node size is proportional to the number of publications, while the thickness of the edges represents the intensity of collaboration (link strength).

The analysis reveals that the 15 most prolific researchers account for 902 of the 2149 publications identified during the study period, with a cumulative 23,762 citations. At the dyadic level, the strongest co-authorship relationships, as measured by Salton’s similarity index, are summarized in Table S2 (Supplementary Materials). Within the network structure, Dr. Guillermo Schmeda-Hirschmann stands out as the most central node (purple cluster), exhibiting the strongest connections with Dr. Cristina Theoduloz (60 co-authored works), Dr. Felipe Jiménez-Aspée (link strength = 29), and Dr. Alberto Burgos-Edwards (link strength = 20) (Figure 4; Table S3, Supplementary Materials).

Dr. Simirgiotis (green cluster) also serves as a key node for articulation, connecting multiple research groups. Among his most prominent collaborations are those with Dr. Bórquez (37 co-authored works) and Dr. Areche (27 co-authored works). The latter, in turn, maintains close collaboration with Dr. Sepúlveda (32 co-authored works), who also collaborates with Dr. Bórquez, thereby forming well-defined research communities and consolidated research lines. Another relevant node is Dr. Carlos Céspedes (yellow cluster), who shows a strong association with Dr. Julio Alarcón (37 co-authored works), while the latter is connected to Dr. Edgar Pastene (link strength = 16), forming an additional cluster. Nonetheless, structural fragmentation is evident in the network: these main cores (represented in pink and green) exhibit little interaction with one another and remain distant from the third axis of relevance, corresponding to the Céspedes–Alarcón cluster (yellow). Dr. Carla Delporte leads the blue cluster together with Drs. Garrido, Backhouse, Muñoz, García, and Pedreschi, integrating phytochemical characterization, pharmacology, metabolomics, and modeling to valorize native Chilean plants against inflammatory pathologies. The light-blue cluster, led by Drs. Fuentes and Palomo focus on the antiplatelet activity and endothelial protection provided by plant-derived bioactive compounds. Finally, the red cluster lacks a clearly defined leader, but Dr. Gloria Montenegro stands out for her prominent collaborations with Dr. Ady Giordano (link strength = 21) and Dr. Raquel Bridi (link strength = 9), forming an additional group within the overall network structure.

### 2.3. Global Collaboration Networks in the Field of Natural Products and Related Topics

**Figure 5:**
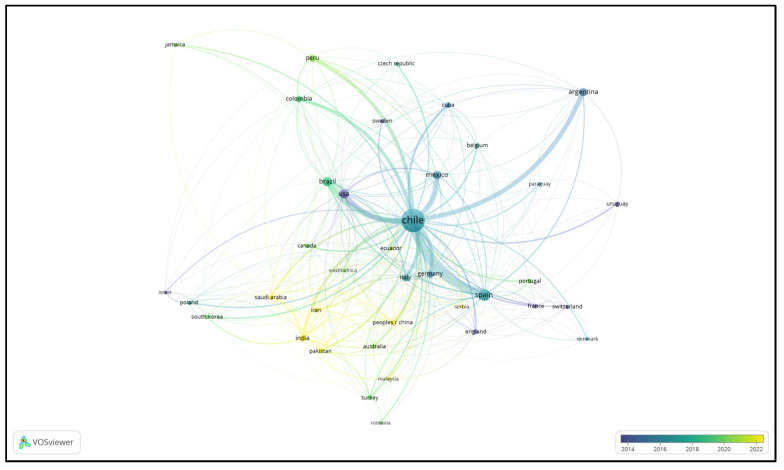
Country-level co-authorship network based on bibliographic data. Node size is proportional to the number of publications, and link thickness reflects the total link strength between countries.

The country-level co-authorship network shows that Chile functions as a central node of international collaboration, establishing its strongest ties with Spain (link strength = 326), followed by Brazil (link strength = 149), the United States (link strength = 131), Mexico (link strength = 121), and Argentina (link strength = 116). This pattern suggests a combination of regional and intercontinental collaboration, with a strong connection to the Ibero-American sphere prior to 2020. More recently, these links have expanded to Asian countries such as India, Pakistan, Iran, Saudi Arabia, and China (Figure 5).

### 2.4. Leading Institutions in the Field of Natural Products and Related Topics

The scientific production on natural products in Chile exhibits a hierarchical and specialized structure. The University of Chile unequivocally leads in both output and impact, with 379 publications and the highest cumulative citation count, establishing itself as the main national reference in the field. At the second level of productivity, the University of Concepción stands out with 221 publications, followed by the University of Talca with 189. Although the latter has a lower publication volume than the preceding institutions, it shows a greater impact in terms of citations per article, evidencing high scientific efficiency and research lines with strong international visibility.

**Figure 6:**
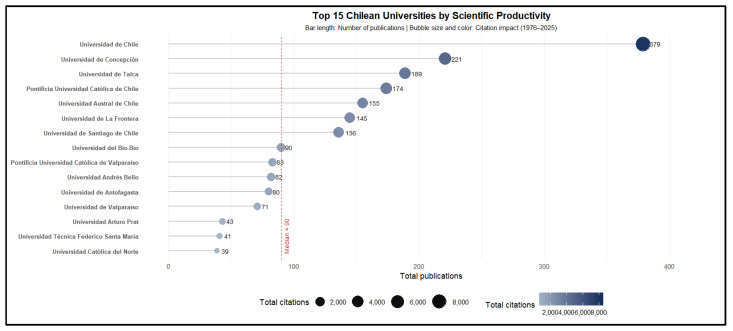
Top 15 Chilean institutions in phytochemistry and natural products by publications and citations (1976–2025). Institutional names are shown in their official Spanish language to preserve their formal designation.

For its part, the Pontifical Catholic University of Chile maintains a balance between productivity and impact, reaffirming its role as a central actor in the research ecosystem. Finally, a “long tail” is observed among regional universities, including the University of La Frontera, the University of Austral Chile, and several institutions in northern Chile. Although these institutions have lower publication output, they make a significant contribution through niches focused on local biodiversity, reflecting active regional participation in the decentralization and thematic diversification of Chilean phytochemistry (Figure 6).

The structure of the Chilean university system, as reflected in the density map, reveals a pronounced core–periphery configuration, in which the University of Chile and the Pontifical Catholic University of Chile act as the gravitational center of the national network. Both institutions not only show the most intense heat spots but also consolidate their leadership through strong collaboration (link strength = 57), functioning as the country’s main engines of research. This metropolitan core naturally includes the University of Santiago (USACH), whose close relationship with the University of Chile (link strength = 44) reinforces the capital’s scientific predominance.

This central dominance is nevertheless balanced by the University of Concepción, which stands out as an independent density pole and a critical regional reference; its critical mass of researchers demonstrates remarkable autonomy from the center, supported by a strategic alliance with the University of La Frontera (link strength = 41). In this landscape, the case of the University of Talca is particularly noteworthy, as it shows a clearly defined heat spot in the lower part of the graph. This density suggests deep specialization in specific areas, such as phytochemistry, enabling it to compete in productivity terms with larger institutions through a diversified collaboration network that includes the University of Chile (link strength = 30), the University of Concepción (link strength = 15), and Andrés Bello University (link strength = 15). Finally, the geographic periphery of the map, comprising institutions such as the Universidad Técnica Federico Santa María and the University of Bío-Bío, displays fainter, more isolated heat spots. This peripheral position suggests a younger scientific output, highly specific research niches, or collaboration networks that, although present, are less dense than those of the consolidated nodes in the central core (Figure S4).

### 2.5. Specialized Journal Core in Natural Products and Related Topics

The 2149 analyzed publications were distributed across 531 journals. The first third of the output was concentrated in just 13 journals, whereas the second and third thirds required 59 and 459 journals, respectively. This pattern evidences a strong concentration of scientific production in a small core of journals, consistent with Bradford’s law. Likewise, the Top 10 journals accounted for 28.71% of all articles, confirming a high degree of editorial centralization followed by a wide dispersion across peripheral journals (Figures S5 and S6).

**Figure 7:**
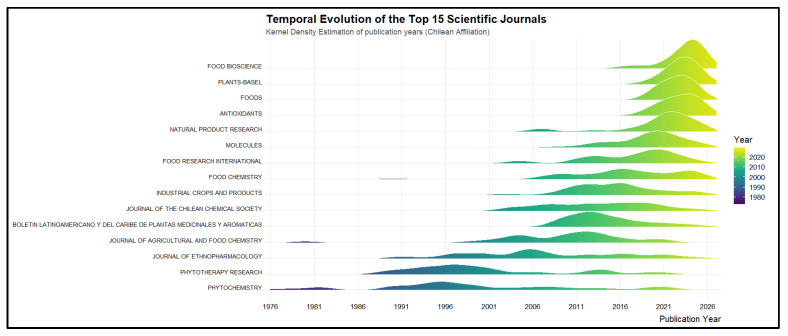
Top 15 scientific journals in Chilean phytochemistry by publication volume and citation impact (1976–2025).

The kernel density plot shows the temporal distribution of publications from 1976 to 2025 across the 15 most relevant journals in phytochemistry and natural products affiliated with Chilean institutions. The color intensity and the height of the curves represent periods of peak research activity, enabling visualization of the evolution of the national scientific dissemination ecosystem (Figure 7).

Iconic journals such as *Phytochemistry* (Elsevier) and *Phytotherapy Research* (Wiley) show sustained activity from the late 1970s through the 1980s, whereas the *Journal of Ethnopharmacology* (Elsevier) consolidated its presence from the 1990s onward. These titles constitute the historical foundation on which the discipline has developed in the country.

**Figure 8:**
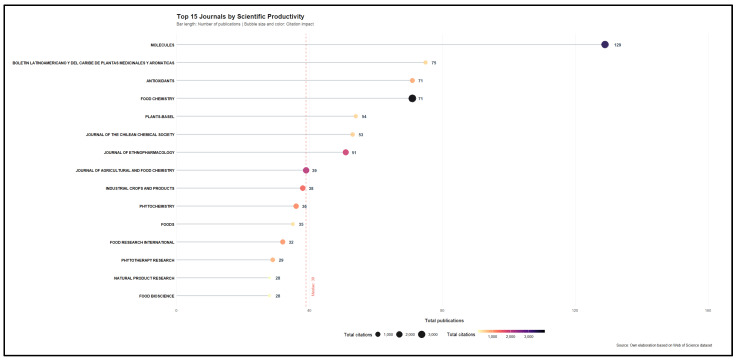
Number of papers published and total citations received by leading scientific journals over the 1976–2025 period.

Over the past two decades, there has been a marked transition from the journals mentioned in the previous paragraph toward open-access, high-output journals such as *Molecules* (MDPI), *Antioxidants* (MDPI), *Foods* (MDPI), and *Frontiers in Pharmacology* (Frontiers), as well as Elsevier’s hybrid journals, including *Food Bioscience* and *Food Research International*. By contrast, the *Journal of the Chilean Chemical Society* (Sociedad Chilena de Química) and the *Boletín Latinoamericano y del Caribe de Plantas Medicinales y Aromáticas* (BLACPMA, MS-Editions) show notable peaks of activity between 2006 and 2016, serving as key platforms for consolidating the regional scientific community prior to the progressive migration toward global journals with higher impact and top-quartile rankings, such as *Food Chemistry* (Q1) (Figure 8).

The analysis of publication output by journal reveals a high concentration of articles in *Molecules*, while *Plants* (Basel) shows the highest publication density in the most recent period. In both cases, authorial leadership is exercised by Mario J. Simirgiotis, who ranks as the researcher with the most publications on these platforms at the national level (Table S4, Supplementary Materials). This dynamic reinforces the field’s transition toward a high-throughput, open-access publication model, in which the rapid dissemination of high-resolution analytical results has become a dominant editorial priority.

**Figure 9:**
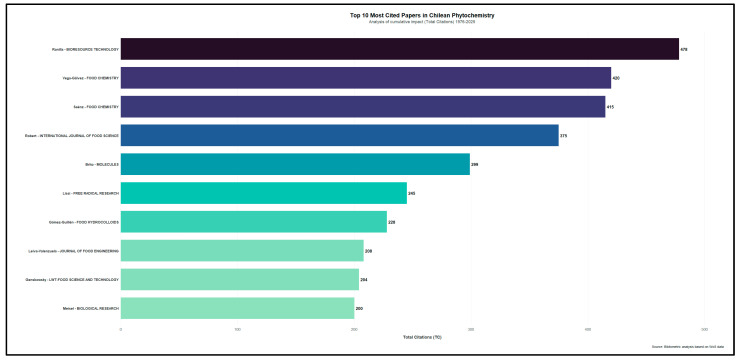
Most prolific scientific publications over the period 1976–2025, ranked by citation count in the Web of Science Core Collection.

The core of scientific impact in Chilean phytochemistry between 1976 and 2025 shows a clear transition from traditional chemical characterization, a demanding task with low editorial visibility, yet essential for establishing the chemical fingerprint of species, to applications in food technology and functional health. The most influential studies, conducted by Luis G. Ranilla and Alejandro Vega-Gálvez, demonstrate that the value of native flora is not limited to its chemical composition, but also extends to its stability under industrial processes such as drying or encapsulation, as well as to its bioactive potential, particularly as antioxidants and antibacterial agents (Figure 9).

This citation pattern confirms that national research has positioned Chilean plant species at the frontier of emerging areas such as materials science and advanced nutrition. In addition, there is a clear predominance of studies focused on phenolic compounds and antioxidant capacity, consolidating this line as the main axis of scientific impact in the field.

### 2.6. Keyword Analysis in Natural Products and Related Topics

A Zipf-type rank–frequency analysis was performed on the unified term corpus, using indexed keywords when available. The logarithmic relationship between term rank and occurrence frequency was modeled by linear regression on a log scale to estimate the Zipf exponent. The rank–frequency distribution showed a highly consistent fit to Zipf’s law, with an exponent of γ = 0.985 and a coefficient of determination of R^2^ = 0.983, indicating a highly organized lexical structure that closely approximates ideal Zipfian behavior. This pattern shows that the corpus is heavily concentrated in a small set of highly frequent terms, whereas most words appear with low frequency, consistent with the classical Zipf distribution (Figure S7).

**Figure 10:**
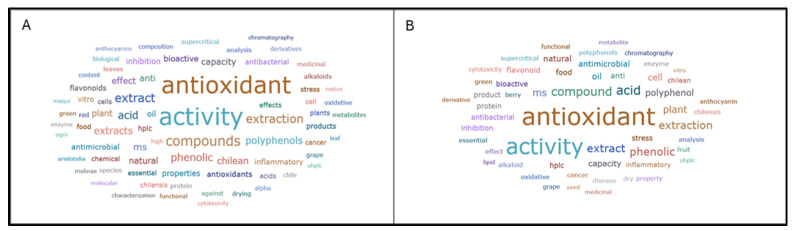
Frequency of terms in titles (**A**) and keywords (**B**) in Chilean phytochemistry research (1976–2025). On the left, a word cloud was generated from the titles of 2149 publications, based on 22,891 analyzed terms. On the right, a word cloud was generated from keywords, considering a total of 18,475 terms. In both cases, the size of each term is proportional to its frequency of occurrence.

The word cloud for the full period from 1976 to 2025, built from the titles of 2149 articles, generated a total of 22,891 words, evidencing the thematic consolidation of Chilean phytochemistry around the bioactivity of natural compounds (Figure 10). The term *antioxidant* (447) emerges as the dominant axis, followed by *activity* (422), confirming that the discipline has evolved toward a focus on the functional evaluation of metabolites (Figures S8–S11). In this context, *extract* (284) and *extraction* (160) underscore the methodological importance of compound isolation, reinforcing a research logic centered on plant extracts and their subsequent biological validation. The co-occurrence of terms such as *phenolic* (131), *polyphenols* (153), *compounds* (184), and *bioactive* further underscores the central role of phenolic compounds as the main drivers of these activities.

The word cloud, constructed from 18,475 keywords indexed in WoS from 1991, the initial year of its indexing, to 2025, evidences the consolidation of Chilean phytochemistry around the bioactivity of natural compounds. The term *antioxidant* (465) emerges as the dominant axis, followed by *activity* (409), confirming the discipline’s functional orientation and showing strong correspondence with term frequencies observed in titles (Figure 10).

The high frequency of terms such as *extract* (162), *extraction* (158), *compound* (180), and plant (129) reflects the centrality of plant extracts and their chemical constituents as units of study. Likewise, the prominent presence of *phenolic* (161), *polyphenol* (114), *flavonoid* (81), and *anthocyanin* (48) indicates that phenolic compounds constitute the main research focus. In parallel, the appearance of terms such as *antimicrobial* (92), *antibacterial* (68), *inhibition* (67), *cytotoxicity* (40), and *inflammatory* (62) evidences a diversification in the biological activities evaluated. Finally, the inclusion of analytical techniques such as *HPLC* (68), *UHPLC* (35), and *MS* (128) represents a methodological advancement, enabling more precise chemical characterization.

The term-frequency treemap, based on a corpus of abstracts from the 2149 publications comprising 439,214 words, reveals a thematic structure concentrated in a limited set of highly recurrent terms. Notably, extract, activity, and use stand out, reflecting a functional sequence centered on the extraction, evaluation, and application of plant-derived compounds (Figure S12). At the same time, the field is organized around a chemical-biological axis dominated by phenolic compounds and antioxidant activity, with strong emphasis on experimental validation. Taken together, these patterns indicate that Chilean phytochemistry is oriented toward molecular characterization and its biological applications. The distribution of Web of Science categories (WC) and Subject Categories (SC) reveals a markedly interdisciplinary structure in Chilean phytochemistry, where organic chemistry, molecular biosciences, and technological applications converge (Figures S13 and S14). The leadership in *Food Science & Technology* and *Chemistry* reflects a dual orientation toward the structural characterization of compounds and the scale-up of these compounds for functional food applications. In turn, the strong presence of *Biochemistry & Molecular Biology* and *Pharmacology & Pharmacy* reflects an emphasis on biological validation and the therapeutic potential of secondary metabolites. The relevance of *Plant Sciences* confirms the central role of plant resources as the foundation of the field.

### 2.7. Researchers’ Perceptions of the Factors Shaping the Development of Chilean Phytochemistry

The interview analysis reveals an ambivalent assessment of phytotherapy in Chile. Although most experts recognize its therapeutic potential and cultural relevance, they also identify multiple structural barriers that hinder its consolidation as a scientific discipline within the health system and national biomedical research.

First, a strong issue of scientific legitimacy emerges. Several researchers agree that phytotherapy could represent a valid alternative in certain health conditions, especially as a therapeutic complement; however, they note that its development in Chile lacks robust clinical validation. They emphasize the absence of Phase I, II, and III clinical trials for most species used, as well as the lack of comprehensive monographs that include pharmacological, toxicological, and comparative efficacy studies. As the researcher (Researcher 1, 2025) notes, “*people do not trust it much, but not because of phytotherapy itself, rather because there are many loose ends that need to be tested in vitro, in vivo, through clinical testing, and through pharmacological trials I, II, and III, which most plants do not have*.” This weakness directly affects perceptions of efficacy and effectiveness, both within the medical community and among project-evaluation bodies, thereby hindering access to competitive funding and hospital integration. This cautious stance is also reflected in the view of Researcher 2 (2025), who states, “*I would not say that it ‘is’ but rather that it ‘could be’ in some cases*,” highlighting a prudent assessment of its therapeutic effectiveness and reinforcing the structural skepticism present in part of the scientific community.

Second, a persistent regulatory and institutional deficit is evident. Experts point to the absence of a clear legal framework and specific public policies to guide and promote the development of national phytotherapy. As one researcher emphasizes, “*the ISP asks you to complete a procedure, called the control regime to be applied, and that procedure ends with them usually telling you that this is either a drug or a food, but there is no intermediate category, and that must happen for Chile to develop*” (Researcher 3, 2025). Although there have been isolated advances, such as the recognition of certain traditional herbal medicines, the system still lacks a comprehensive framework that links basic research to health regulation and technology transfer. This situation generates profound legal uncertainty, limiting product standardization and hindering the translation of scientific results into clinical or industrial applications, thereby preventing the field’s academic maturity from yielding value-added innovations.

A third relevant element is disciplinary fragmentation. There is a disconnect among chemists, botanists, foresters, agronomists, biologists, pharmacologists, and clinicians. Some experts warn that natural product chemists often focus on structural elucidation without delving deeply into pharmacology or biology, whereas pharmacologists have rarely worked directly with the plant itself. This fragmentation is not only scientific but also structural, and it needs to be addressed through policies that promote effective interdisciplinary integration. In this context, one interviewee noted that “*there are a large number of scientists actively working on the protective effects of natural products in the context of biomedicine, but these efforts often lack the necessary support to grow, consolidate, and move beyond the laboratory*” (Researcher 7, 2025). Likewise, another researcher stated that “*strengthening support among researchers and creating spaces so that the knowledge generated actually reaches medical practice is essential for phytotherapy to stop being seen as a marginal practice and begin to occupy a legitimate, evidence-based place within our health system*” (Researcher 23, 2025).

Likewise, the absence of formal training in phytotherapy within health science programs constitutes a significant structural barrier. Most interviewees agree that phytotherapy is not part of medical curricula or other health-related disciplines, which fosters professional unfamiliarity and, consequently, skepticism. In this regard, Researcher 10 (2025) notes that “*most health professionals do not even consider them; that is, they are not shown in training systems or at universities*.” This educational gap contributes to the fact that many conventional medical practitioners do not regard phytotherapy as a legitimate therapeutic option or perceive it as a practice lacking sufficient evidence. This situation is reinforced by the comparative perception noted by Researcher 15 (2025), who states that “*in Chile many people (I would say the majority) do not take it into account, but in other countries it is often used in a complementary way*,” suggesting a relative lag in the national context compared with international models in which integration is greater.

At the sociocultural level, important tensions are also identified. Although the use of medicinal plants is deeply rooted in Chilean culture, especially in rural contexts and family traditions, several experts point to a progressive loss of traditional knowledge associated with urbanization. At the same time, phytotherapy is described as being stigmatized as a practice associated with historically marginalized social sectors or with rural settings, which may generate prejudice in academic and clinical environments dominated by the conventional biomedical model. In this sense, there is also an epistemological gap between the dominant biomedical model, centered on single molecules and standardized clinical trials, and the multicomponent, contextual, and traditional nature of phytotherapy.

Another factor that hinders the field’s development is the tension between scientific research and commercialization. Some interviewees warn that the market tends to promote herbal products through marketing strategies that lack scientific grounding, fostering distrust among the public and professionals and potentially compromising public health. In this regard, one expert describes the sector’s future “*as a growing, unserious, and potentially dangerous business for health*” (Researcher 2, 2025). This researcher also provides evidence of the risks of uncontrolled commercialization, particularly through e-commerce and open-air markets, noting that the premature sale of preparations without adequate standardization harms the field’s reputation and reinforces perceptions of a lack of rigor. According to this interviewee, there is still no safety control in place, except perhaps interventions related to the presence of rodents or insects in sites where medicinal plants are stored (Researcher 2, 2025).

Taken together, the testimonies show that phytotherapy in Chile is at an early stage of development, characterized by substantial therapeutic and cultural potential but limited structural integration. The barriers identified extend beyond the lack of specific studies and instead constitute a scientific, institutional, and sociocultural framework that will shape the field’s future consolidation as a research domain and formal health practice.

### 2.8. The Future of Phytotherapy Research in Chile

The qualitative analysis revealed that phytotherapy in Chile occupies a structurally ambiguous position, characterized by weak articulation among traditional knowledge, scientific production, and the formal healthcare system. As one interviewee noted: “*There is no integration of phytotherapy into the healthcare system in Chile. It simply does not exist*” (Researcher 18, 2025). On the one hand, the widespread use of phytotherapy has spurred numerous in vitro studies to validate beneficial properties and substantiate the ethnomedicinal relevance of certain plant species. On the other hand, few studies have progressed to preclinical models, and even fewer have advanced to clinical trials. Although the number of publications has increased, institutional integration remains limited, thereby constraining both the scope of many studies and the sector’s tangible impact on the healthcare system.

Researchers identified four main structural barriers to the development of phytotherapy: (1) insufficient training within the healthcare sector, (2) inadequate standardization and quality validation, (3) regulatory rigidities that constrain scaling and innovation, and (4) limited and fragmented state action lacking a comprehensive strategic framework. These barriers not only hinder clinical integration and access to therapeutic alternatives but also shape the national scientific landscape by perpetuating a fragmented research model that relies on individual initiatives and has limited capacity for technology transfer. As one interviewee noted, it is essential that “studies do not remain solely at the level of basic research, where a paper is produced, published, and the process ends there” (Researcher 20, 2025), but instead advance toward real-world applications.

The lack of systematic training in phytotherapy within health-related degree programs constitutes a critical gap. As another interviewee stated: “*There are no study programs… it is not as though one can train as a pharmacist or physician with formal knowledge in phytotherapy*” (Researcher 18, 2025). There are no established curricula or professional pathways that enable informed prescribing, safety monitoring, or appropriate clinical guidance. This absence not only limits clinical integration but also constrains the capacity to develop applied research with tangible health impact, thereby perpetuating the disconnect between academic production and medical practice.

The current challenges are further compounded by the lack of reliable information on native plant species, which exhibit a high degree of endemism. It is imperative to establish modern quality control standards that accurately determine the qualitative and quantitative composition of each batch throughout the entire value chain. This gap in chemical standardization and traceability directly undermines the sector’s credibility.

In this context, weaknesses can be identified in the scientific evidence underpinning the current regulatory framework (DS3/2010). Beyond the regulation’s formal basis, inconsistencies persist in validating the properties attributed to various species. A paradigmatic example is boldo (*P. boldus*), whose technical monograph highlights anti-inflammatory and hepatoprotective effects based primarily on preclinical evidence; however, the existence of sufficient clinical evidence cannot be substantiated, particularly given the date of the educational and technical transfer documents. This lack of rigor extends even to the Chilean Pharmacopeia, where significant inaccuracies have been identified, such as the attribution of apomorphine—a synthetic compound—as the active principle of boldo, thereby conflating it with the naturally occurring aporphine-type alkaloids characteristic of the species.

The regulatory dimension thus emerges as a critical frontier. The national control system mandates that plant-derived products be classified either as “phytopharmaceuticals” or “foods,” with no intermediate category to accommodate preventive products or standardized traditional uses. As noted during fieldwork: “*You are typically told that this is either a drug or a food, but there is no intermediate category*” (Researcher 18, 2025).

Historically, in Chile, Supreme Decree No. 286 (2001) established the category of Traditional Herbal Medicines (THMs), defining them as artisanal products labeled in accordance with popular tradition. However, this regulatory framework has effectively become a “sacrosanct text,” remaining largely unchanged through to the current Technical Standard No. 133 (2012). When the Ministry of Health was consulted regarding the criteria for including plant species, Official Communication CP No. 4001 indicated that the list originated from a working group convened in Santiago (the country’s capital) in 1991, with supporting evidence limited to five general-interest publications. Sector stakeholders have questioned this centralized origin. As one interviewee noted: “*The THM list was developed solely with the participation of herbal vendors from La Vega Poniente (a traditional market in Santiago)… it reflects practices from the central region rather than other areas of Chile*” (Researcher 23, 2025).

The rigidity of the system prevents its timely updating, as noted by one informant: “*Incorporating a plant into the Technical Standard requires its formal amendment, which entails a prioritization process that, to date, has not taken place*” (Researcher 13, 2025). Within this regulatory gap, processes of product registration, investment, and scaling are significantly hindered. In this way, regulation not only structures the market but also delineates the forms of innovation that are viable within the country and which types of knowledge attain institutional legitimacy.

In parallel, a critical public health concern emerges regarding informal commercialization. The proliferation of purported “miracle cures” and untraceable mixtures, distributed through both physical markets and e-commerce channels, significantly increases the risk of pharmacological interactions and undermines the credibility of the rational use of phytotherapy. In this context, the challenge extends beyond strictly technical considerations; a comprehensive health governance strategy is required, one that integrates public scientific literacy with the effective strengthening of regulatory oversight mechanisms.

From a prospective standpoint, most experts anticipate that, in the short to medium term, efforts will remain largely fragmented, with progress concentrated on a limited number of species—primarily those with greater public recognition and strong roots in the country’s biocultural heritage—driven by increasing levels of public–private collaboration, as many already have established markets. These advances are expected to be led by universities with a critical mass of researchers. Nonetheless, skepticism persists regarding the likelihood of deep structural transformations. However, a global resurgence of phytotherapy is also acknowledged, along with a significant strategic opportunity (Table 1). The scientific development of medicinal species—particularly native ones—could contribute to cultural identity, territorial development, and job creation, provided it is grounded in robust evidence, rigorous quality standards, and a coherent regulatory governance framework.

In this context, frontier technologies play a central role not only in knowledge generation but also in reducing regulatory asymmetries. High-resolution chromatographic and spectrometric platforms, such as UHPLC–QTOF–MS, have transformed chemical characterization capabilities, enabling the establishment of objective standards for identity, purity, and concentration that facilitate the standardization and traceability processes required by regulatory agencies.

Tools such as metabolomics, GNPS, and molecular networking not only enhance the systematic identification of metabolites but also provide a robust scientific basis for the multicomponent complexity of plant extracts—an essential consideration for their regulatory recognition as therapeutic systems rather than undefined mixtures. Likewise, in the context of functional validation, approaches such as high-throughput screening, advanced cellular models, transcriptomics, proteomics, network pharmacology, and machine learning enable the multitarget complexity of natural products to be addressed from an integrative perspective. In parallel, strategies such as nanoformulation and integrated drug discovery platforms help improve bioavailability, safety, and technological scalability. These approaches not only accelerate knowledge generation for individual species but also reduce the scientific uncertainty that hinders regulatory processes, thereby becoming strategic instruments for public policies aimed at the sustainable valorization of medicinal biodiversity.

Finally, interviewees characterize the future of phytotherapy as a landscape shaped by political economy factors. As one participant noted, “*It is perceived as a competitor to the revenue generation associated with commercial pharmaceuticals*” (Researcher 20, 2025). This perception positions phytotherapy as a potential rival to the traditional pharmaceutical industry, generating economic tensions that may marginalize it, even where therapeutic or preventive value exists. Without shifts in incentive structures, increased public investment in applied R&D, the development of specialized human capital, and regulatory adjustments that create viable pathways for standardization, integration is likely to remain slow and uneven.

## 3. Discussion

Phytochemical research in Chile has experienced sustained expansion over recent decades, as evidenced by an annual growth rate of 11.46% and a doubling time of 6.4 years. This trend positions the country within the global movement toward the revalorization of natural products as a source of innovation in pharmaceutical, nutraceutical, and biotechnological applications, and confirms the consolidation of phytochemistry as a strategic field within the national scientific system [38,39,40]. These findings indicate that phytochemistry in Chile is undergoing rapid expansion. This prospective trend suggests that the study of natural resources has reached a level of institutional maturity that ensures its scalability and continued relevance within the national research agenda. The predominance of terms such as “activity” and “antioxidant” in titles and keywords indicates that the Chilean scientific agenda has been strongly oriented toward the functional validation of bioactive metabolites, particularly those associated with plant species used in traditional medicine [30,41,42]. This trend is consistent with the global shift from descriptive approaches to bioactivity-centered studies, in which the identification of secondary metabolites is driven not only by pharmacological interests but also by regulatory requirements for the standardization of herbal products [3,43,44,45].

The observed thematic evolution reveals a transition from an initial stage focused on chemotaxonomy and structural isolation to a phase of functional characterization based on antioxidant assays and biological models, culminating in a recent stage dominated by high-resolution analytical tools, metabolomics, green synthesis, and integrated computational chemistry approaches [46,47,48]. More recently, phytochemical research has increasingly incorporated artificial intelligence and data-driven methodologies, expanding the field’s analytical and translational capabilities. Machine learning and deep learning techniques are being applied to medicinal plant identification, the analysis of high-dimensional omics datasets, metabolite discovery, structural elucidation, and the characterization of complex phytochemical profiles. At the same time, the development of interoperable datasets integrating taxonomic, chemical, and ethnobotanical information is creating new opportunities for biodiversity monitoring, authentication of medicinal species, and natural product research [49,50]. Artificial intelligence is also contributing to natural product-based drug discovery through virtual screening, molecular interaction prediction, and the identification of novel bioactive scaffolds, strengthening the links between phytochemistry, pharmacology, and biotechnology. These developments reflect an increasing convergence between phytochemistry, metabolomics, biodiversity informatics, and computational sciences, broadening both the methodological scope of the discipline and its potential applications in medicine, agriculture, and biotechnology [51,52,53]. This methodological shift suggests an effort to advance and a level of disciplinary maturation consistent with international standards, although a strong reliance on preclinical and laboratory-based studies still persists [54,55].

From the perspective of scientific networks, the co-authorship structure reveals a highly centralized system, characterized by a limited number of highly productive authors with extensive collaborative ties, alongside subgroups with strong internal cohesion but limited interconnection. This pattern is consistent with the values observed for the Salton index and the Association Strength index, which indicate the coexistence of a dominant core and specialized clusters with relatively closed linkages. The cluster led by the Universidad de Talca group constitutes the most influential axis in terms of biological validation and collaborative articulation [56,57], whereas other nodes, such as Simirgiotis [58,59,60], Areche [61,62], or Céspedes and Alarcón [63,64,65], contribute specialized expertise in advanced chemical analysis and metabolomic fingerprinting. However, the limited interconnections among these groups may constrain knowledge circulation, reduce interdisciplinary synergies, and hinder the emergence of more integrative research lines.

A particularly relevant finding is the persistent gap between basic and applied scientific productivity. Although Chile demonstrates a strong capacity for the identification, isolation, and characterization of secondary metabolites, this strength is not proportionately reflected in clinical development, regulatory approvals, or derived pharmaceutical products [37,45]. This interpretation is further supported by several indicators associated with the technological and translational maturity of the national phytopharmaceutical sector (Supplementary Table S5, Supplementary Materials). Clinical validation remains limited, with fewer than 10 identified clinical studies involving Chilean affiliations, most of which focus on dietary supplementation rather than phytotherapeutic development. Likewise, fewer than 20 related patents were identified, and among the 112 phytopharmaceutical products currently recognized in the national regulatory framework, only 3 are based on native Chilean species (*P. boldus*, *B. globosa*, and *Haplopappus* sp.). Additional evidence is provided by the limited integration of phytotherapy into the public healthcare system, as reflected in the limited availability of scientifically validated phytopharmaceuticals for healthcare professionals and the low representation of native medicinal species in the formal regulatory framework. Furthermore, the industrial ecosystem remains relatively small, comprising approximately 15 companies, predominantly small and medium-sized enterprises, while the export profile of medicinal non-wood forest products is still largely concentrated on low-value-added raw materials, such as boldo leaves, quillaja bark, and crude extracts, rather than standardized phytopharmaceuticals or locally developed pharmaceutical innovations. Together, these indicators suggest that the Chilean system continues to operate predominantly at early stages of technological maturity, constraining the clinical, industrial, and commercial translation of the scientific knowledge generated [37,45].

Similarly, the semantic analysis reveals weak integration between phytochemical research and ecological sustainability. Concepts such as conservation, sustainable management, and biodiversity are only marginally represented, despite Chile having a highly vulnerable endemic flora exposed to extractive pressures. This disconnect is critical, as it compromises the future viability of the biological resource and reinforces the need to adopt integrated frameworks such as One Health, which link human health, ecosystem conservation, and the sustainable use of medicinal species [37,66,67]. Moreover, the growing scientific and commercial interest in bioactive metabolites may increase harvesting pressure on wild populations if it is not accompanied by strategies for sustainable resource management, cultivation, and domestication. In this context, strengthening the integration among phytochemical research, conservation biology, and public policy is essential to ensure that the valorization of native biodiversity aligns with long-term ecosystem protection and sustainable harvesting practices [37,67].

In parallel, a structural tension emerges between biodiversity and regulation. The Chilean regulatory framework requires quality, safety, and traceability standards that are difficult for small producers and traders to meet, thereby perpetuating high levels of informality in the herbal market. These barriers are not only reflected in stakeholder perceptions but are also consistent with the current regulatory framework governing traditional herbal medicines. In particular, the Chilean Technical Standard recognizes only a limited subset of traditionally used species and restricts their accepted therapeutic indications to predefined categories, leaving little room to incorporate emerging scientific evidence or to formally recognize additional native medicinal species. Although this regulatory approach is intended to protect public health, it may also act as a structural barrier to local innovation and to the incorporation of phytotherapeutic products into the formal healthcare system, especially when it is not accompanied by technical support policies, scientific validation, and technology transfer [16,17,37,45].

Beyond the bibliometric indicators, the development of phytochemical research in Chile has been strongly supported by a relatively small group of native medicinal species that have become reference models for pharmacological, biotechnological, and translational studies. Species such as *P. boldus*, *Q. saponaria*, *B. globosa*, and *A. chilensis* have attracted sustained scientific interest due to their diverse bioactive compounds, including alkaloids, saponins, phenylpropanoid glycosides, anthocyanins, and polyphenols, with reported applications ranging from hepatoprotection and wound healing to vaccine adjuvants and functional foods. At the same time, emerging research on species such as *Cryptocarya alba* (Molina) Looser [Lauraceae], *Drimys winteri* J.R.Forst. & G.Forst. [Winteraceae], *Laurelia sempervirens* (Ruiz & Pav.) Tul. [Atherospermataceae], and *Haplopappus* spp. [Asteraceae] highlights the untapped potential of Chilean native biodiversity. Despite remarkable advances in phytochemical characterization and biological evaluation, most of these species face common challenges in pharmacological validation, chemical standardization, safety and pharmacokinetic assessment, technological development, and clinical translation. A summary of representative species, their principal bioactive compounds, current applications, and future research opportunities is presented in Tables S6–S15 (Supplementary Materials).

Recent years have also witnessed the incorporation of advanced biotechnological approaches into Chilean phytochemical research. These include high-resolution metabolomics and chemical fingerprinting, HPLC-ESI-QTOF-MS and LC-MS-based analytical platforms, supercritical fluid extraction, microencapsulation technologies, the development of *Quillaja* saponin-based vaccine adjuvants, green synthesis of nanoparticles, and, more recently, the integration of computational approaches and artificial intelligence for medicinal plant identification, metabolite discovery, and phytochemical data analysis. Together, these advances are expanding the translational and industrial potential of Chilean native medicinal species while opening new opportunities for innovation and sustainable biotechnology.

Another notable phenomenon is the editorial shift toward highly visible open-access journals, particularly those published by MDPI, such as *Molecules* and *Plants*. This migration reflects both the internationalization of Chilean scholarly output and broader structural changes in the scientific publishing ecosystem [30,68,69]. However, it also exposes researchers to rising publication costs, creating new inequalities in access to scientific dissemination. In Latin American contexts, where research budgets are limited, this economic pressure may influence publication strategies and journal selection [30,68,69].

Methodologically, the combination of bibliometric analysis and qualitative evidence provides a more comprehensive view of the field than approaches based exclusively on quantitative indicators [34,70]. Triangulation made it possible to identify structural gaps, such as collaborative fragmentation, the absence of translational capacity, and weak ecological integration, which do not emerge clearly from conventional bibliometric indicators. This mixed-methods approach constitutes a strength of the study and offers a replicable framework for analyzing emerging scientific systems [34].

Among the limitations, it should be acknowledged that reliance on Web of Science underrepresents non-indexed output, grey literature, and non-formalized traditional knowledge, which is particularly relevant in ethnobotanical contexts [71,72]. Nevertheless, the analyzed corpus adequately captures the dominant currents of formal academic research and enables robust trends in disciplinary evolution to be identified. In addition, challenges associated with author name disambiguation may affect the structure of co-authorship networks and collaboration metrics.

It is a critical challenge for bibliometric studies to work with search strings on the Web of Science platform because the limited number of terms that can be combined with Boolean operators reduces the search string’s coverage and sensitivity [73,74]. This restriction is especially relevant in cross-cutting research areas such as medicinal plants and phytochemistry. In these fields, articles are often titled only with the name of the active compound or the species’ scientific name, omitting key generic terms such as phytochemicals or medicinal plants. This taxonomic and chemical fragmentation in titles may lead to an underestimation of the available literature if the search string does not adequately capture the field’s terminological diversity.

In summary, Chilean phytochemistry has reached a level of scientific maturity and methodological sophistication; however, its future outlook depends on overcoming several critical structural challenges: breaking the insularity among scientific hubs, strengthening translational research, and integrating sustainability into the analytical agenda. Ultimately, the success of the field will not be measured merely by the identification of new metabolites, but by its ability to connect laboratory-based knowledge with health regulation and clinical development, thereby transforming native biodiversity into therapeutic innovation and sustainable public value.

The testimonies show that phytotherapy in Chile is in its early stages of development, characterized by high therapeutic and cultural potential but low structural integration. The barriers identified are not limited to the absence of specific studies; rather, they reflect a scientific, institutional, and sociocultural framework that conditions the future consolidation of this field of research and formal healthcare practice. In summary, the future of phytotherapy in Chile depends not only on traditional knowledge or isolated scientific promise, but on the country’s capacity to align applied science, coherent regulation, and the healthcare system within a governance framework that integrates biodiversity, technological innovation, and health sovereignty, ensuring responsible, scientifically validated, and sustainable use.

Beyond the historical contributions of researchers affiliated with Chilean institutions, our findings indicate that phytochemistry and medicinal plant research in Chile has progressively adopted an increasingly biotechnology-oriented approach. This transition has been characterized by the implementation of high-resolution analytical techniques, including HPLC, LC-MS/MS, GC-MS, NMR, metabolomic fingerprinting, and chemometric tools, which have substantially improved the characterization, standardization, and quality control of native medicinal flora. These advances have facilitated the characterization and identification of bioactive compounds, promoting the development of high-value applications such as phytopharmaceuticals, nutraceuticals, functional foods, biopesticides, and vaccine adjuvants. However, our analysis also shows that, for emblematic species such as *Q. saponaria* and *P. boldus*, institutions with foreign affiliations, highlighting important opportunities to strengthen national research capacity and innovation, have conducted a considerable proportion of the most advanced research and biotechnological applications.

Despite these significant technological advances, our analysis indicates that translational research has not progressed at the same pace. Critical gaps remain in clinical validation, the development of sustainable cultivation systems for large-scale medicinal biomass production, advanced formulation technologies, and metabolomic traceability, as well as in the integration of computational methods, artificial intelligence, network pharmacology, and multi-omics approaches. Collectively, these findings suggest that future research should strategically focus on bridging advanced phytochemical characterization with applied biotechnology, sustainable production, and clinical validation. Furthermore, our results highlight the need for a coordinated national strategy to unlock the biotechnological potential of Chilean native medicinal plants, particularly endemic species such as *P. boldus* and *Q. saponaria*. Such a strategy should promote long-term investment in species-specific research, sustainable cultivation, value-added product development, and technology transfer, thereby enabling the full utilization of Chile’s remarkable biodiversity and high level of endemism.

Bibliometric studies have an important methodological limitation: a comprehensive analysis of medicinal plants requires species-specific search strategies that use each species’ scientific name along with its principal phytochemical markers and bioactive compounds. A clear example of this limitation is the case of Dr. Bruce Cassels, a distinguished medicinal chemist, whose scientific output was not fully captured by the search algorithm. Consequently, the magnitude of his contributions to Chilean phytochemistry is not fully reflected in our analysis (Table S16, Supplementary Materials). Nevertheless, the main strength of bibliometric studies lies in their ability to provide a macro-level overview of research trends, enabling the identification of strengths, knowledge gaps, and emerging opportunities. This explains the large number of bibliometric studies published each year. Therefore, to understand why much of the research has not successfully progressed from the preclinical stage to clinical validation, it is necessary to complement this approach with mixed-methods studies, such as the one conducted in the present work.

## 4. Materials and Methods

### 4.1. Mixed Methods: Bibliometric and Qualitative Analysis

A sequential explanatory mixed-methods design was used, in which the first stage consisted of a quantitative bibliometric analysis, followed by a complementary qualitative phase to deepen and interpret the identified structural findings. In the quantitative phase, performance analysis and scientific mapping techniques were applied to examine the evolution, productivity, impact, and interrelationships of the field of study. Specifically, core bibliometric indicators were employed, including Price’s, Lotka’s, Bradford’s, and Zipf’s laws, as well as Hirsch’s *h*-index.

In the second stage, an inductive content analysis was conducted to capture key stakeholders’ perceptions. To this end, in-depth interviews were conducted, and the resulting data were analyzed using an inductive approach. This triangulation made it possible to assess the thematic coherence of the corpus and to contextualize the emerging conceptual clusters from the standpoint of practice and the researchers’ perspectives.

### 4.2. Data Source and Search Strategy

The bibliographic information was retrieved from the Web of Science Core Collection (WoS), selected for its rigorous indexing criteria, multidisciplinary coverage, and recognition as a primary source in international bibliometric studies.

A structured search strategy using Boolean operators was designed to carry out two searches aligned with the study objectives. No initial restrictions were applied regarding publication year or document type, except those inherent to the thematic search strategy. The searches were conducted on 18 March 2026. The following thematic search strings were used: (a) Ts = ((extract) OR (isolated) AND (plant)), then filtered by article and country (Chile); (b) TS = ((medicinal NEAR/0 plant) OR (natural NEAR/0 product) OR (phytochem*)) AND CU = (Chile). As an inclusion criterion, only documents classified as articles were considered. The thematic search tag (TS) retrieves records from the title, abstract, author keywords, and Keywords Plus^®^ fields, whereas the CU tag restricts the results to documents with at least one author affiliated with an institution in the specified country.

The first search string (a) yielded 871 records, and the second (b) yielded 4847 records. The bibliometric data collection phase concluded with the consolidation of a master file containing 5718 records, exported from Web of Science in Plain Text format under the “Full Record and Cited References” option. This technical configuration was essential to ensure the integrity of the metadata required for subsequent mathematical modeling. From this corpus, a matrix of bibliometric variables was constructed, with variables selected strategically based on their analytical utility.

Productivity and Authorship (AU, AF, UT): The use of authorship fields, together with the unique identifier, enabled the modeling of Lotka’s law, allowing the distribution of productivity to be assessed and the most influential authors in the field to be identified.

Temporal Evolution and Growth (PY): The publication year was used as an independent variable to verify compliance with Price’s law, determining the annual growth rate and the maturity stage of the phytochemical field.

Dispersion and Sources (SO): The Source variable was processed to apply Bradford’s law, thereby allowing journals to be ranked into specialization cores based on their thematic relevance.

Thematic and Semantic Analysis (DE, ID, TI, AB): Author and Keywords Plus^®^ terms, together with natural language processing of titles and abstracts, formed the basis for Zipf’s law, identifying the conceptual core and the hierarchy of dominant terms.

Impact and Collaboration Networks (TC, NR, C1, WC, SC): Times cited and cited references were used to measure the impact and currency of knowledge, while affiliations (C1) and subject categories (WC/SC) facilitated the mapping of institutional decentralization and the sector’s interdisciplinarity.

This set of metadata enabled both performance analysis and scientific mapping.

#### Data Processing and Standardization

To ensure the accuracy of productivity and impact indicators, a manual and algorithmic normalization protocol was implemented for the authorship field (AU). Given that international bibliometric databases often fragment the identities of researchers with compound surnames or spelling variants, a hybrid search engine was developed in the R language.

This procedure made it possible to unify key figures such as Alarcón-Enos, J. (often incompletely indexed as Alarcón, J.) and Céspedes, C. (who appears in variants such as Céspedes-Acuña), as well as to consolidate authors with high variability in their registration or international indexing, such as Schmeda-Hirschmann, G., Simirgiotis, M., and Vega-Gálvez, A. A critical aspect of this quality control was the use of regular-expression-based exclusion filters to eliminate bibliometric “noise” from homonyms. This filtering made it possible to accurately distinguish the output of the selected group leaders from that of other researchers with similar surnames and affiliations. This approach ensures that the citation and co-authorship metrics presented reflect the actual productivity of the research hubs analyzed, avoiding the distortions common in strictly automated bibliometric studies.

### 4.3. Initial Filtering and Technical Cleaning of Publications

#### 4.3.1. Methodology: Selection and Consolidation of the Corpus

The study selection process was structured into four successive phases—identification, screening, assessment, and consolidation—using a standardized technical exclusion protocol to ensure the integrity of the corpus.

#### 4.3.2. Identification and Initial Cleaning

Through consolidated searches in the Web of Science platform, 5718 records were initially identified. After removing duplicates (*n* = 150) and excluding records outside the primary thematic scope (*n* = 130), a total of 5438 documents were retained for screening.

#### 4.3.3. Screening and Abstract Review

During the screening phase, a thorough review of titles, keywords, and abstracts was conducted. To ensure the accuracy of the process, 80% of abstracts from the preselected documents were analyzed, allowing thematic relevance to be confirmed when the title was ambiguous. At this stage, 3258 records were excluded for failing to meet the defined inclusion criteria, leaving 2180 potentially relevant documents.

#### 4.3.4. Third-Level Review and Validation

The selected records underwent full-text evaluation, during which 31 technical inconsistencies were identified, including borderline cases and indexing errors. These disputed records were then referred to the extended research team for third-level review. Through an arbitration and consensus process based on a critical reading of the articles, the discrepancies were resolved, and the records were excluded for failing to strictly meet the established methodological criteria.

It is important to note that, following the initial retrieval of records, an individual manual review was conducted for publications associated with highly prolific researchers, since their scientific output spans multiple domains of natural product research beyond plant phytochemistry. This procedure enabled the identification and exclusion of studies that did not meet the predefined inclusion criteria: original research articles published in English and focused on plant species. For example, in the case of Dr. Guillermo Schmeda-Hirschmann (University of Talca), publications dealing with animal-derived natural products were excluded, such as ‘The Parotoid Gland Secretion from Peruvian Toad *Rhinella horribilis* (Wiegmann, 1833): Chemical Composition and Effect on the Proliferation and Migration of Lung Cancer Cells’, ‘Antiproliferative activity and new argininyl bufadienolide esters from the “cururú” toad *Rhinella* (Bufo) *schneideri’*, ‘Cytotoxicity and antimitotic activity of *Rhinella schneideri* and *Rhinella marina* venoms’, and ‘A Paraguayan toad *Rhinella schneideri* preparation based on Mbya tradition increases mitochondrial bioenergetics with migrastatic effects dependent on AMPK in breast cancer cells’. Likewise, studies related to fungi or fungal-derived metabolites were excluded, including ‘Antimicrobial Butyrolactone I Derivatives from the Ecuadorian Soil Fungus *Aspergillus terreus* Thorn. var. terreus’, ‘Metabolites from *Microsphaeropsis olivacea*, an endophytic fungus of *Pilgerodendron uviferum’*, ‘Cycloaspeptide A and pseurotin A from the endophytic fungus *Penicillium janczewskii’*, and ‘Antifungal activities of extracts produced by liquid fermentations of Chilean *Stereum* species against *Botrytis cinerea* (grey mould agent)’. Records corresponding to other document types or disciplinary areas were also removed, including the review article ‘Traditional medicine and gastroprotective crude drugs’ and the synthetic chemistry study ‘Synthesis of tricyclic analogs of stephaoxocanidine and their evaluation as acetylcholinesterase inhibitors’. Similarly, for Dr. Javier Echeverría Morgado (University of Santiago de Chile), manual screening enabled the exclusion of publications incorrectly classified as original articles, such as ‘Edible fruits and berries as a source of functional polyphenols: current scene and future perspectives’ and ‘Natural products attenuate PI3K/Akt/mTOR signaling pathway: A promising strategy in regulating neurodegeneration’, both of which are review articles. These examples illustrate that the construction of the final corpus did not rely exclusively on automated record retrieval but incorporated expert validation for each publication, thereby reducing false positives and strengthening the thematic specificity and methodological robustness of the bibliometric database.

#### 4.3.5. Definitive Corpus

**Figure 11:**
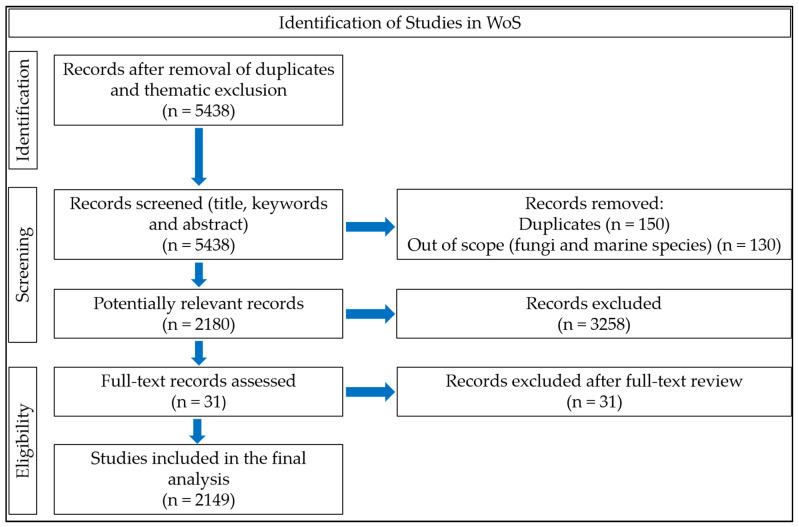
Workflow diagram for the selection of records in Web of Science (WoS) for a bibliometric analysis.

As a result of the protocol, a final corpus of 2149 publications was consolidated for the definitive analysis (Figure 11; Table 2).

### 4.4. Analysis and Visualization

The bibliometric analysis was structured into two complementary levels: performance analysis and scientific mapping. Data processing was carried out in the R (v4.4.1; R Foundation for Statistical Computing, Vienna, Austria) programming environment and the RStudio interface (Posit Software PBC, Boston, MA, USA), specifically using the Bibliometrix package (v4.3.2) and its web-based application, Biblioshiny (https://www.bibliometrix.org; accessed on 5 June 2026), to conduct the database analyses. For the construction of bibliometric maps based on co-authorship matrices, VOSviewer version 1.6.20, from the Centre for Science and Technology Studies at Leiden University, was used, enabling a multidimensional exploration of the scientific corpus. ATLAS.ti (v25; Scientific Software Development GmbH, Berlin, Germany) was used for text analysis [75,76,77].

#### 4.4.1. Performance Analysis

Classical bibliometric indicators were applied to assess the structural dynamics of the field:

Price’s law, to analyze the temporal growth of scientific output and assess its exponential behavior. Lotka’s law, to examine the distribution of scientific productivity among authors. Bradford’s law identifies the core set of journals that concentrate the largest share of publications.

The Zipf’s law analysis was conducted in R using a cleaned corpus derived from the combined Author Keywords (DE) and Keywords Plus (ID) fields retrieved from the Web of Science database and previously processed in ATLAS.ti. Additional preprocessing involved removing whitespace, punctuation, alphanumeric codes, numerical values, very short terms, and generic stop words unrelated to the thematic content. The cleaned terms were then grouped and ranked by frequency of occurrence. Next, logarithmic transformations of term frequency and rank were computed (log_10_ frequency versus log_10_ rank), and a linear regression model was fitted to evaluate the extent to which the data conformed to Zipf’s law. Finally, the scale exponent (γ) and the coefficient of determination (R^2^) were obtained from the regression model [78].

Additionally, Hirsch’s h-index was used to measure the relative impact of the corpus and key authors.

Likewise, traditional descriptive indicators were analyzed, including: (1) the annual publication trend; (2) the most productive journals and their evolution; (3) the authors with the highest contributions and their evolution; and (4) the institutions with the greatest contributions and their evolution over the study period.

#### 4.4.2. Relational Analysis (Scientific Mapping) Examined the Collaborative Structure of the Field Through Co-Authorship Analysis Among Authors, Institutions, and Countries

The collaborative structure of the field was examined through a co-authorship analysis of authors, institutions, and countries, treating the joint appearance of these actors in the same document as a co-occurrence event. Network normalization was performed using Salton’s index (cosine similarity), a method selected for its ability to reduce biases associated with differences in absolute publication frequency among researchers. Minimum occurrence and citation thresholds were also applied to enhance analytical robustness and improve the visual clarity of the resulting graphs.

Cluster identification was carried out using VOSviewer’s clustering algorithm, with prior normalization based on Salton’s index to quantify collaboration strength [79,80].

Following Glänzel (2001), this measure represents the network’s “natural topology” by normalizing co-authorship links, thereby facilitating the identification of thematic and professional specialization cores that might otherwise be obscured by the raw publication volumes of leading authors [81]. This methodology enabled mapping the structure of the field in Chilean phytochemistry, distinguishing consolidated scientific cores from peripheral networks with lower relational density [79,81].

#### 4.4.3. Sensitivity Analysis of the Search

To further evaluate the robustness of the bibliometric search strategy and estimate the potential impact of relying on broad thematic descriptors, a sensitivity analysis was performed using *P. boldus* as a sentinel species. This species was selected because it is one of the most extensively studied native Chilean plants and has a broad range of scientific applications, including phytochemistry, pharmacology, ecology, agriculture, biotechnology, and industrial uses. Owing to its high publication volume and thematic diversity, *P. boldus* provides a suitable and conservative model for assessing potential retrieval losses associated with topic-based bibliometric searches.

A complementary search was conducted in the Web of Science database using the species’ scientific name exclusively. The retrieved records were subsequently processed and compared with the main bibliometric database using DOI-based matching and duplicate removal procedures implemented in R. After excluding publications already included in the original bibliometric corpus, an additional set of relevant records was identified. These publications were manually reviewed and classified according to their predominant translational level or principal area of application. The complete list of these articles is provided in Supplementary Table S16.

The additional records displayed a consistent indexing pattern. Most titles were structured around the scientific name of the species (*Peumus boldus*), the name of specific metabolites (e.g., boldine), particular biological activities (e.g., antimicrobial, insecticidal, antioxidant, antiproliferative, or anti-inflammatory), or specific application domains (e.g., essential oils, nanotechnology, non-timber forest products, and grain protection). In contrast, broad descriptors such as “medicinal plant,” “phytochemistry,” or “phytochemical compounds” were not explicitly represented in the retrieved titles.

These findings indicate that a proportion of the scientific literature on natural products and phytochemical research may be indexed primarily by species names, individual compounds, or application-specific terminology rather than by broad thematic descriptors. Consequently, bibliometric strategies based exclusively on general topic keywords may not fully capture the extent of the available literature. In this context, the present sensitivity analysis serves as a complementary methodological exercise to evaluate potential retrieval bias and provide additional evidence for the robustness and consistency of the bibliometric approach adopted in this study.

#### 4.4.4. Estimation of Future Publications

To estimate the future evolution of phytochemical research in Chile, an exponential growth model based on Price’s law was applied to the annual publication series. Annual publication counts were log-transformed and fitted using a log-linear regression model, where the slope coefficient represents the annual growth rate. The model showed a strong goodness of fit (R^2^ ≈ 0.95), yielding an estimated annual growth rate of 11.46% and a doubling time of approximately 6.4 years. The projected number of publications by 2035 (674 articles) was obtained by extrapolating the fitted exponential trend to that year, assuming the current growth pattern remains stable.

### 4.5. Qualitative Study

A qualitative study was conducted using semi-structured interviews and document review to examine developments in phytochemistry and medicinal plants and to explore how the field is expected to evolve [82,83,84,85].

The interviews were conducted both in person and online, with audiovisual recording obtained prior to informed consent [86]. In addition, respondents were given the option of completing the instrument in writing, a format used by 20% of the experts due to scheduling constraints.

#### 4.5.1. Participant Selection

A sample of expert researchers was identified using the following selection criteria: holding a doctoral degree and having more than 10 scientific publications. Seventy-one researchers were contacted via their institutional email addresses, yielding 25 responses from PhD holders with extensive experience in medicinal chemistry in Chile, drawn from both the public and private sectors.

#### 4.5.2. Participant Selection and Sampling Strategy

Participant selection was conducted using a criterion-based purposive sampling strategy to recruit information-rich cases with extensive knowledge and experience in natural products and phytochemistry. Eligible participants were identified based on documented research trajectories, including scientific publications, participation in research and innovation projects, and recognized expertise in the chemical characterization and application of natural resources. To capture a broad range of perspectives, the sample was intentionally designed to maximize disciplinary and institutional diversity, incorporating researchers from universities, public institutions, and private companies working in fields such as phytochemistry, medicinal chemistry, ethnobotany, biology, biochemistry, non-timber forest products (NTFPs), and related areas. (Table 3).

#### 4.5.3. Recruitment Criteria

Potential participants were identified through a review of scientific publications, research and innovation projects, institutional websites, and professional networks related to phytochemistry, medicinal plants, and natural products research in Chile. Eligible individuals were invited by e-mail using their publicly available institutional contact information and were provided with information regarding the objectives and scope of the study. Participation was voluntary, and all interviewees provided informed consent prior to data collection, in accordance with the ethical approval granted for the study.

#### 4.5.4. Data Collection, Triangulation, and Ethical Considerations

The interviews and document review were conducted in 2025 and 2026. Data were collected through in-depth interviews conducted either orally or in written format, depending on participant availability and preference. Most interviews were audio-recorded and subsequently transcribed verbatim, while approximately 25% were completed in writing and returned electronically via email. The documentary review included scientific articles, academic theses, technical documents, and the current regulatory framework.

Methodological and source triangulation were applied by integrating information from interviews and documentary sources, enabling the comparison of complementary perspectives and strengthening the credibility and validity of the analysis.

All participants signed informed consent forms, and confidentiality was ensured by assigning alphanumeric codes. The Scientific Ethics Committee of Universidad Mayor (Record No. 0595) approved the study.

### 4.6. Qualitative Data Analysis

The qualitative data were analyzed using a conventional inductive content analysis approach. The analytical corpus comprised verbatim transcripts of audio-recorded interviews, written interview responses submitted electronically, and documentary sources included in the review. Each interview and document constituted a unit of analysis.

The analysis began with repeated readings of the corpus to achieve deep familiarization with the data and to identify emerging patterns and recurring ideas. In the initial stage, the material was organized into preliminary thematic areas for exploration, after which units of meaning were identified, condensed, and transformed into interpretive codes [86]. Coding and data management were conducted using ATLAS.ti v25, which facilitated the systematic organization of the material and ensured traceability throughout the analytical process.

Through an iterative process of constant comparison, similar codes were progressively grouped into broader subcategories and analytical categories, leading to the development of explanatory dimensions related to the motivations, practices, risks, and determinants associated with the use of plant species for medicinal purposes [86]. Data collection and analysis proceeded concurrently until thematic saturation was achieved, that is, when no new conceptually relevant categories emerged from the data [86].

To enhance the rigor and trustworthiness of the analysis, methodological and source triangulation were applied by integrating evidence from interviews and documentary sources. In addition, the coding framework and emerging categories were reviewed and discussed by the research team throughout the analytical process until interpretive consensus was reached.

### 4.7. Integration of Results (Mixed-Methods Approach)

The quantitative results derived from the performance analysis and scientific mapping were integrated with the findings of the qualitative analysis.

This integration made it possible to:

- Validate the thematic coherence of the identified clusters.
- Reduce conceptual noise.
- Contextualize the structural patterns observed.
- Identify research gaps and opportunities.

The quantitative-qualitative triangulation strengthened the study’s methodological consistency and deepened the interpretation of the results.

## 5. Conclusions

This study, based on a sequential mixed-methods design that integrates a bibliometric analysis with interviews with key actors in the field, characterizes and explains the development of phytochemical and phytotherapeutic research in Chile from 1976 to 2025. From a quantitative perspective, the results show a gradual consolidation of research on natural products, as reflected in sustained growth in scientific output, the emergence of academic schools, and the publication of their work in high-visibility international journals. However, this development has not been proportionally translated into clinical advances, technological scaling, or the generation of validated phytotherapeutic products, revealing a persistent gap between basic research and its sanitary and industrial applications.

The qualitative phase helps explain the causes of this gap. Researchers’ accounts indicate that the field is marked by a strong sociocultural appreciation of medicinal plants, but also by weak integration into the health system, disciplinary fragmentation, and structural limitations in clinical validation, regulation, and professional training. In addition, risks associated with uninformed use were identified, including self-medication, lack of traceability, and the mistaken perception that natural products are inherently safe.

Taken together, both approaches show that the development of phytotherapy in Chile does not depend solely on scientific output, but on a broader system in which scientific, regulatory, institutional, and sociocultural factors interact. In this context, regulation emerges as a critical element, not only for safeguarding public health but also for facilitating or constraining innovation, technology transfer, and sectoral formalization.

Despite these limitations, the results point to a significant strategic opportunity. Native biodiversity, ethnomedicinal knowledge, and advances in technologies such as metabolomics and integrative pharmacology place Chile in a favorable position to develop products with territorial identity and added value. However, capitalizing on this opportunity requires stronger coordination among academia, the State, the health system, the productive sector, and communities that hold traditional knowledge, under a One Health approach.

In this sense, the future of phytotherapy in Chile appears to be one of contingent integration. Its consolidation will depend on public policies that strengthen applied research, train specialized human capital, and modernize regulation, accompanied by incentives for intellectual property protection. Among the main challenges are developing a national herbal pharmacopeia and transitioning scientific clusters from academic co-authorship to effective co-innovation networks, thereby enabling the creation of intermediate regulatory categories and strengthening technical support mechanisms to formalize the sector.

In summary, the study shows that phytotherapy in Chile is in a transitional phase, marked by high cultural legitimacy and low institutionalization. Bridging this gap will require moving from a fragmented system toward an integrated and sovereign model in which scientific evidence, regulation, public health, and the protection of traditional heritage converge to ensure the safe, effective, and sustainable use of medicinal plants.

## Figures and Tables

**Table 1:** Current configuration and projection of the phytotherapy system in Chile: scientific, institutional, and sociocultural determinants.

Dimension	Level of Influence	Present	Future or Necessary Condition
Scientific Evidence	Direct (scientific–technical)	Predominance of in vitro and phytochemical studies, with limited preclinical and clinical validation.	Development of preclinical and clinical trials, generation of robust evidence, and therapeutic validation.
Quality and Composition	Direct (scientific–technical)	High variability in product quality, without standardization of botanical identity or concentration.	Implementation of quality standards (GMP), composition control, and traceability.
Pharmacopoeia and Technical Standards	Direct (scientific–technical)	Fragmented, incomplete, or outdated references for many species.	Development of a national herbal pharmacopoeia with complete monographs and quality criteria.
Biodiversity and Development	Direct (scientific–technical)	High richness of native medicinal flora, underutilized in research and development.	Systematic study of native species and development of value-added products with territorial identity.
Research and Development	Direct (scientific–technical)	Mostly basic research, with limited transfer to products or policies.	Promotion of applied research, technological scaling, and linkage with industry and the state.
Therapeutic Role	Direct (scientific–technical)	Primarily complementary use, without consolidated clinical validation.	Integration is a validated complementary practice, with evidence, safety, and controlled use.
Public Health Risks	Direct (scientific–technical)	Self-medication, misidentification errors, pharmacological interactions, and lack of control.	Strengthening of pharmacovigilance, traceability, and education in safe use.
Regulation	Structural (institutional–productive)	Fragmented framework, rigid categories (drug/food), and limited oversight.	Development of clear regulatory frameworks, intermediate categories, and strengthened oversight.
Role of the State	Structural (institutional–productive)	Emerging participation, with partial advances in regulation and funding.	Active state as regulator, funder, and articulator of public policies under a One Health approach, strengthening academia’s participation in scientific evaluation processes, phytochemical validation, and technical advisory on plant species with medicinal use.
Integration into the healthcare system	Structural (institutional–productive dimension)	Limited, localized, and non-structural integration within primary healthcare (PHC) or other settings.	Gradual integration into the healthcare system, particularly within PHC, supported by established protocols and supervision.
Professional training	Structural (institutional–productive dimension)	Absence of systematic training in phytotherapy within health-related degree programs.	Formal, certified, and interdisciplinary training in phytotherapy.
Healthcare professionals	Structural (institutional-productive)	Limited incorporation into clinical practice, associated with lack of knowledge or skepticism.	Enhanced clinical training and capacity for informed prescribing of phytotherapeutic agents.
Market and productive sector	Structural (institutional–productive dimension)	Presence of an informal market, heterogeneity in product quality, and poorly regulated commercialization.	Market formalization, traceability, quality control, and the development of productive value chains.
Social use and motivations	Contextual (sociocultural)	Widespread use driven by tradition, accessibility, cost, and trust in natural remedies.	Transition toward informed, critical use supported by health education.
Perception of the natural	Contextual (sociocultural)	Association between “natural,” harmless, and safe, fostering uncritical use.	Public education on risks, active metabolites, and interactions.
Culture and traditional knowledge	Contextual (sociocultural)	Ancestral knowledge remains current, but with only partial recognition and limited integration.	Protection of knowledge, institutional recognition, and strengthening of intercultural models.
Territorial dimension (urban–rural)	Contextual (sociocultural)	Greater use in rural areas; in urban settings, use is more market-mediated.	Differentiated strategies by territory, integrating local knowledge and regulation.
Intercultural governance	Contextual (sociocultural)	Limited participation of traditional communities in sector decision-making.	Effective intercultural dialogue and inclusive governance models.
System projection	Cross-cutting synthesis	Incipient, fragmented development, with uneven progress across dimensions.	Consolidation depends on articulation among scientific evidence, regulation, training, investment, and governance.

**Table 2:** Characterization of the document corpus for analysis.

Variable	Value (or Sample, *n*)	Unit	Subsampling Criterion
Documents	2149	Articles	Hirsch’s index (h-index)
Time	1979–2025	Year	Continuous period (no gaps), Price’s Law (1)
Place (Affiliation)	1	Country/Territory	Census
Authors	6878	Persons	Lotka’s Law
Keywords (DE)	6592	Words	Zipf’s Law
Keywords Plus (ID)	4988	Words	Zipf’s Law
Journals	531	Journals	Bradford’s Law

**Table 3:** Informant profiles.

Informant Profiles	Description of the Interviewees
Experts, academics, and researchers	Specialists with experience in the study, chemical characterization, and application of natural resources. This group includes researchers affiliated with Chilean universities, companies, and public institutions, drawn from disciplines such as chemistry, medicinal chemistry, ethnobotany, biology, biochemistry, medicine, and related fields, whose research focuses on phytochemistry, phytotherapy, non-timber forest products (NTFPs), secondary metabolites, medicinal plants, natural dietary supplements, and other plant-derived applications.

## Data Availability

The original contributions presented in this study are included in the article/Supplementary Materials. Further inquiries can be directed to the corresponding authors.

## Supplementary Materials

The following supporting information can be downloaded at: https://www.mdpi.com/article/10.3390/plants15142135/s1, Figure S1. Citation distribution and h-index of the Chilean natural products corpus (1976–2025); Figure S2. Projected annual number of Web of Science publications with Chilean affiliation in phytochemistry for the period 2026–2035; Figure S3. Author Productivity Distribution and Lotka’s Law Fit (1976–2025); Figure S4. Institutional co-authorship density map in Chile; Figure S5. Bradford’s law distribution of journals in Chilean phytochemistry publications (1976–2025); Figure S6. Core sources identified according to Bradford’s Law in Chilean phytochemistry (1976–2025); Figure S7. Log–log rank–frequency distribution (Zipf’s law) of terms extracted from combined Author Keywords (DE) and Keywords Plus (ID) in the retrieved articles (1976–2025); Figure S8. Word cloud showing the most frequent terms extracted from 1371 research titles in the period 2016–2025. The size of the words represents their frequency of occurrence; Figure S9. Word cloud showing the most frequent terms extracted from 514 research titles during 2006–2015. The size of the words represents their frequency of occurrence; Figure S10. Word cloud showing the most frequent terms extracted from 198 research titles from 1996–2005. The size of the words represents their frequency of occurrence; Figure S11. Word cloud showing the most frequent terms extracted from research titles during the early development of Chilean phytochemistry (1976–1995); Figure S12. Treemap of terminological frequencies in Chilean phytochemistry literature (1976–2025); Figure S13. Illustrates the distribution of the 30 most frequent Web of Science categories (WC) among Chilean phytochemistry publications from 1976–2025, using a treemap where rectangle size reflects document counts; Figure S14. Shows the distribution of the 20 most frequent research areas (SC) in Chilean phytochemistry literature (1976–2025), using a treemap where rectangle size corresponds to publication frequency from Web of Science broader subject classifications; Table S1. Top 25 ranking of authors based on productivity and impact; Table S2. Strongest co-authorship relationships according to Salton’s similarity index among Chilean phytochemistry researchers (1976–2025); Table S3. Collaboration of Chilean researchers; Table S4. Top 15 ranking of journals based on productivity and impact. Table S5. Indicators of technological and translational maturity in the Chilean phytopharmaceutical sector. Tables S6–S15. Regulatory status, principal bioactive compounds, current applications, and future research opportunities of *Peumus boldus* Mol.; *Quillaja saponaria* Mol.; *Buddleja globosa* Hope; *Aristotelia chilensis* (Mol.) Stuntz; *Cryptocarya alba* (Mol.) Looser; *Drimys winteri* J.R.Forst. & G.Forst; *Laurelia sempervirens* (Ruiz & Pav.) Tul.; *Haplopappus* spp. (Bailahuén); *Kageneckia oblonga* Ruiz & Pav.; *Schinus latifolius* (Gillies ex Lindl.) Engl. Table S16. *Peumus boldus* publications that were omitted from the topic-based search.
