# Supplementary material for: The Evolution of Phytochemical and Medicinal Plant Research in Chile: Status, Opportunities, and Challenges
Source: Plants (Basel). 2026 Jul 10;15(14):2135. doi: 10.3390/plants15142135 (PMC13415090; doi:10.3390/plants15142135)
Supplement: Supplementary file 1 [file plants-15-02135-s001.zip › plants-4373053-supplementary.pdf]

Supplementary Material

Article

The Evolution of Phytochemical and Medicinal Plant Research in Chile: Status, Opportunities, and Challenges

Gonzalo Fuentes-Barros [1,2], Sebastián Castro-Saavedra [1,3], Nicolás Montalva [4], Alejandro Vega-Muñoz [5,6], Jaime Mella [7,8], Antonia Díaz-Valdés [4], Camila Zoppi [1], Ana Caroline Avanco [2], Marco Mellado [9,10,*] and Javier Echeverría [3,*]

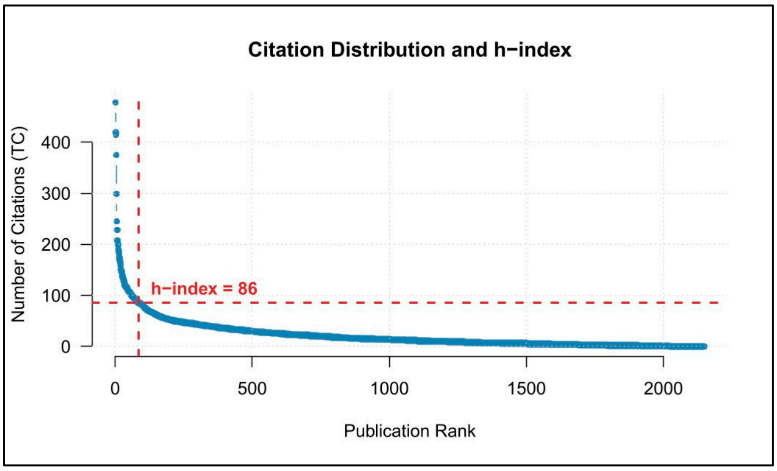

Figure S1. Citation distribution and h-index of the Chilean natural products corpus (1976–2025); the corpus reached an h-index of 86.

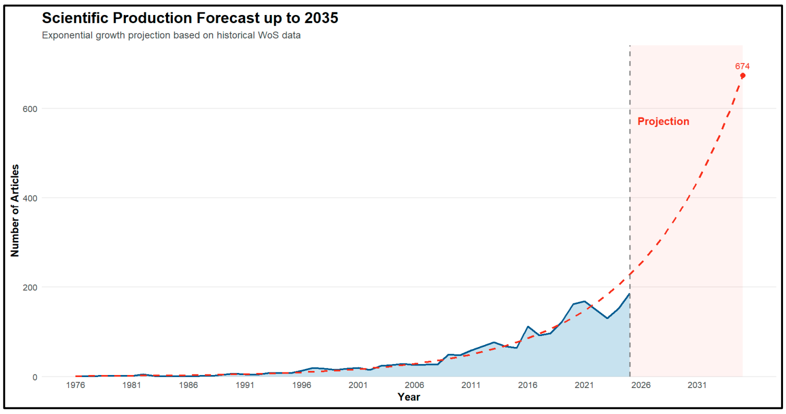

Figure S2. Projected annual number of Web of Science publications with Chilean affiliation in phytochemistry for the period 2026–2035. The red dashed line represents an extrapolation of the exponential growth model based on historical publication trends.

Table S1. TOP 25 Ranking of authors based on productivity and impact.

N°	AU_final	Selected papers according to the methodology			Web of Science profile (*)	
		**Total Papers**	**Total Citations**	**Average citations per paper**	**Total Papers**	**Total Citations**
1	SCHMEDA-HIRSCHMANN G	121	4037	33,36	288	6198
2	SIMIRGIOTIS M	109	2746	25,19	188	3291
3	CESPEDES C	69	1586	22,99	216	3512
4	SEPULVEDA B	66	1816	27,52	57	1102
5	THEODULOZ C	65	2048	31,51	141	3196
6	ALARCON-ENOS J	60	1364	22,73	83	949
7	BORQUEZ J	55	1182	21,49	99	1012
8	MONTENEGRO G	55	1150	20,91	127	1912
9	PAREDES A	54	906	16,78	56	522
10	ARECHE C	51	1237	24,25	99	1718
11	DELPORTE C	49	1665	33,98	57	1151
12	BECERRA J	40	630	15,75	72	781
13	FUENTES E	37	913	24,68	157	3097
14	VEGA A	36	1636	45,44	155	5550
15	PALOMO I	35	846	24,17	196	3912
16	JIMENEZ-ASPEE F	34	756	22,24	62	935
17	PASTENE E	34	780	22,94	81	1406
18	ECHEVERRIA J	33	325	9,85	161	3098
19	FERNANDEZ K	33	865	26,21	77	1492
20	MADRID A	33	351	10,64	98	882
21	MUNOZ O	33	693	21,00	64	788
22	URZUA A	33	902	27,33	157	2047
23	RODRIGUEZ J	30	1161	38,70	51	1340
24	GIORDANO A	28	444	15,86	56	782
25	MARDONES C	27	682	25,26	69	1570

SCHMEDA-HIRSCHMANN G represents the sum of the PBQ-0322-2025 and G-1046-2010 profiles, whereas CESPEDES C represents the sum of the KPC-1004-2024, D-3138-2013, and KYY-9431-2024 profiles. (*) Accessed on 14 June 2026.

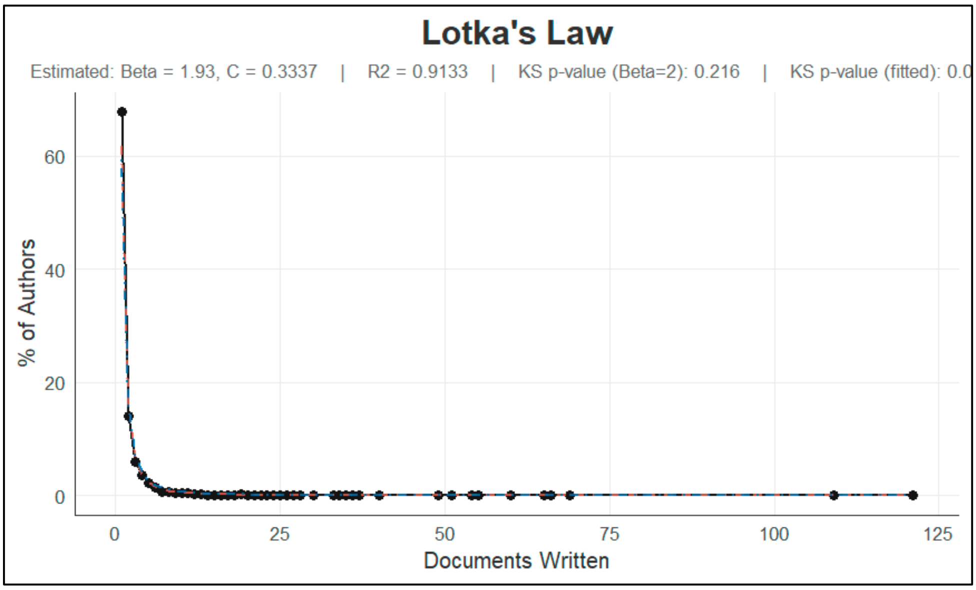

Figure S3. Author Productivity Distribution and Lotka's Law Fit (1976–2025). The scatter plot illustrates the frequency distribution of author productivity within the Chilean phytochemistry corpus on a logarithmic scale. The dashed red line represents the theoretical Lotka distribution model ($y = c / x^k$). The estimated exponent ($k = 1.93$) is close to the classical theoretical value of 2.0, indicating a typical concentration pattern of scientific productivity. The coefficient of determination ($R^2 = 0.913$) demonstrates a strong fit between the observed data and the power-law model. Additionally, the non-significant Kolmogorov–Smirnov test for the theoretical distribution ($p = 0.216$) indicates that the observed distribution does not differ significantly from the expected Lotka pattern.

Table S2. Strongest co-authorship relationships according to Salton's similarity index among Chilean phytochemistry researchers (1976–2025).

Salton	Author Pair
0,861	FUENTES E – PALOMO I
0,708	ARECHE C – SEPULVEDA B
0,677	SCHMEDA-HIRSCHMANN G – THEODULOZ C
0,575	ALARCON J – CESPEDES C
0,535	GIORDANO A – MONTENEGRO G
0,511	JIMENEZ-ASPEE F – THEODULOZ C
0,478	BORQUEZ J – SIMIRGIOTIS M
0,452	JIMENEZ-ASPEE F – SCHMEDA-HIRSCHMANN G
0,394	SEPULVEDA B – SIMIRGIOTIS M
0,362	ARECHE C – SIMIRGIOTIS M
0,34	RODRIGUEZ J – THEODULOZ C
0,282	RODRIGUEZ J – SCHMEDA-HIRSCHMANN G
0,273	ECHEVERRIA J – URZUA A
0,213	BORQUEZ J – SEPULVEDA B
0,203	BORQUEZ J – PAREDES A
0,173	PAREDES A – SIMIRGIOTIS M
0,151	ARECHE C – BORQUEZ J
0,149	DELPORTE C – MUNOZ O
0,119	DELPORTE C – VEGA A
0,111	ALARCON J – PASTENE E
0,1	ECHEVERRIA J – SIMIRGIOTIS M

Table S3. Collaboration of Chilean researchers.

Collaborators	Joint Papers
SCHMEDA - THEODULOZ	60
SIMIRGIOTIS - BORQUEZ	37
CESPEDES - ALARCON	37
ARECHE - SEPULVEDA	32
FUENTES- PALOMO	31
SIMIRGIOTIS - ARECHE	27
SIMIRGIOTIS - SEPULVEDA	27
SIMIRGIOTIS - PAREDES	13
BORQUEZ - PAREDES	10
BORQUEZ - SEPULVEDA	10
ALARCON - FUENTES	10
ALARCON - PALOMO	12

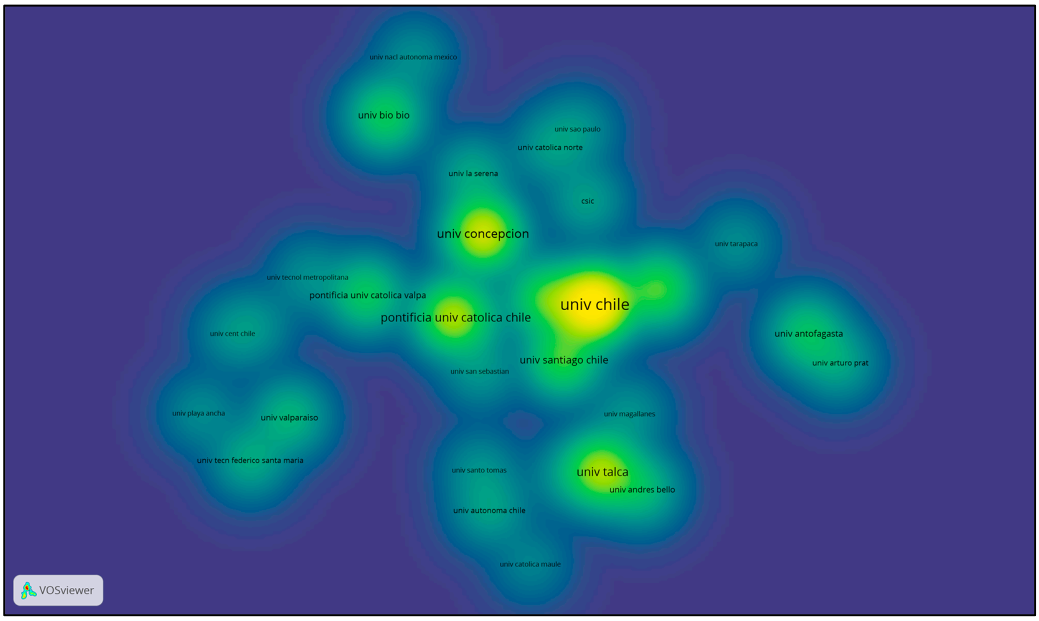

Figure S4. Institutional co-authorship density map in Chile. This visualization represents the concentration of scientific output through density analysis. Areas in bright yellow indicate a higher density of publications and a tighter collaboration network.

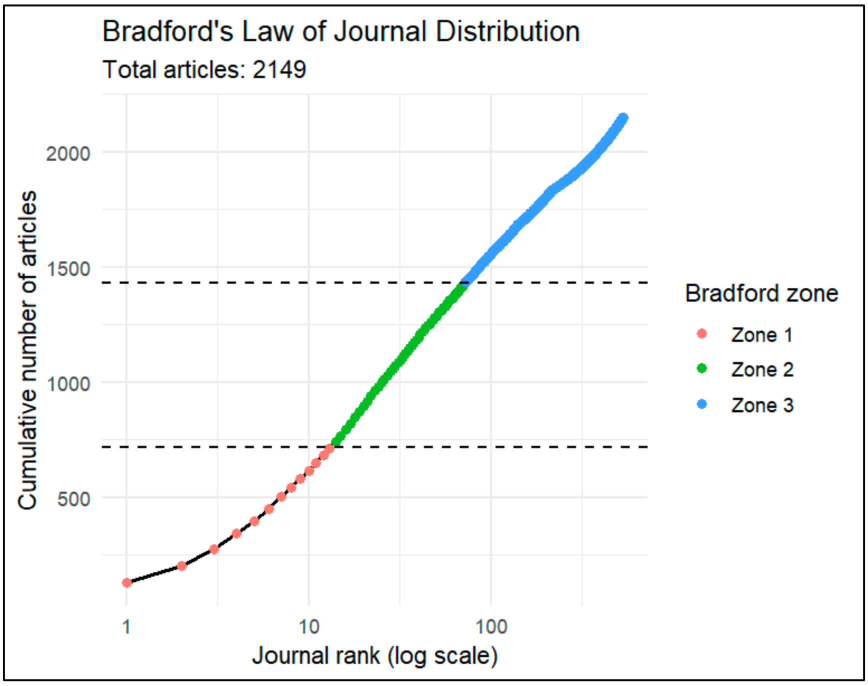

Figure S5. Bradford's law distribution of journals in Chilean phytochemistry publications (1976–2025). Journals are ranked by decreasing productivity on a logarithmic scale.

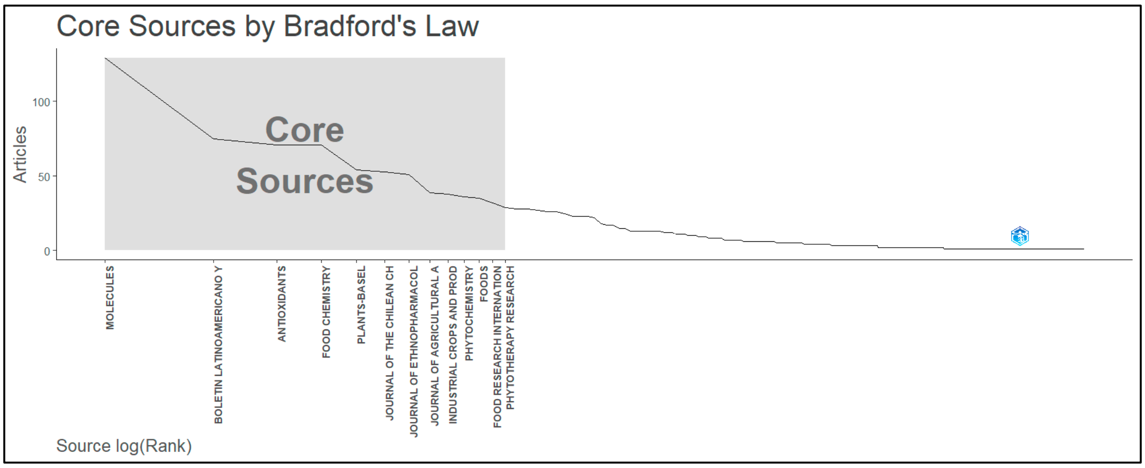

Figure S6. Core sources identified according to Bradford's Law in Chilean phytochemistry (1976–2025). Journals are ranked in decreasing order of the number of articles. The shaded area represents the Bradford core, comprising the most productive sources that account for the largest share of publications, followed by a long tail of less productive journals.

Bradford's distribution identifies *Molecules* and the *Latin American and Caribbean Bulletin of Medicinal and Aromatic Plants* (BLACPMA) as central axes in the dissemination of phytochemical research in Chile. This high concentration within the core (Zone 1) reveals a strategic duality: while *Molecules* provides global reach and a rigorous chemical standard, BLACPMA has consolidated itself as the most relevant regional hub for the visibility of local phytochemistry.

On the other hand, the prominent presence of journals such as *Antioxidants*, *Food Chemistry*, and *Phytochemistry* confirms that national research exhibits a strong orientation toward bioactivity (particularly antioxidant capacity) and food chemistry, which closely aligns with the analysis of the most frequent terms. Finally, the inclusion of high-impact journals such as *Journal of Ethnopharmacology*, *Frontiers in Pharmacology*, *Plants*, *Foods*, and *Industrial Crops and Products* demonstrates that, from the perspective of natural products research, a very broad spectrum of the plant world is addressed, responding with versatility to the multifunctionality and diversity of applications assigned to botanical resources in the country.

The *Journal of the Chilean Chemical Society* and *BLACPMA* are two national journals whose scope includes studies with chemico-medicinal applications, particularly focused on native species with a high degree of endemism. Although their specialized focus may limit their appeal to specific audiences, these journals play a fundamental role in the country's scientific sovereignty, enabling the documentation and valorization of Chilean biodiversity.

Regarding the dispersion analysis, the mean Bradford multiplier was 5.94, indicating a high dispersion of the scientific literature beyond the core. While the second zone required 4.54 times more journals than the core to cover the same volume of articles, the third zone required 7.78 times more titles than the second. This increasing geometric progression reveals a pronounced peripheral expansion, characteristic of a discipline that, while grounded in a solid core, permeates multiple interdisciplinary areas.

Table S4. TOP 15 ranking of journals based on productivity and impact.

Top journals	Total Papers	Corresponding author	Most Cited Paper	Citations (TC)
MOLECULES	129	Simirgiotis, M	HPLC-UV-MS Profiles of Phenolic Compounds and Antioxidant Activity of Fruits from Three Citrus Species Consumed in Northern Chile	299
BOLETIN LATINOAMERICANO Y DEL CARIBE DE PLANTAS MEDICINALES Y AROMATICAS	75	Nieto, A	Anti-inflammatory Activity of *Aristotelia chilensis* Mol. (Stuntz) (Elaeocarpaceae)	35
ANTIOXIDANTS	71	Rubilar, O	Antioxidant Activity as an Indicator of the Efficiency of Plant Extract-Mediated Synthesis of Zinc Oxide Nanoparticles	65
FOOD CHEMISTRY	70	Vega-Galvez, A	Effect of Air-Drying Temperature on Physico-Chemical Properties, Antioxidant Capacity, Colour and Total Phenolic Content of Red Pepper (*Capsicum annuum* L. var. Hungarian)	420
PLANTS-BASEL	55	Pastene-Navarrete, E	Green Extraction of Alkaloids and Polyphenols from *Peumus boldus* Leaves with Natural Deep Eutectic Solvents and Profiling by HPLC-PDA-IT-MS/MS and HPLC-QTOF-MS/MS	68
JOURNAL OF THE CHILEAN CHEMICAL SOCIETY	53	Solís, C	Antibacterial and Antifungal Terpenes from *Pilgerodendron uviferum* (D. Don) Florin	51
JOURNAL OF ETHNOPHARMACOLOGY	52	Schmeda-Hirschmann, G	Traditional Medicine and Gastroprotective Crude Drugs	111
JOURNAL OF AGRICULTURAL AND FOOD CHEMISTRY	39	Schmeda-Hirschmann, G	Free Radical Scavengers And Antioxidants From Lemongrass (*Cymbopogon citratus* (DC.) Stapf.)	189
INDUSTRIAL CROPS AND PRODUCTS	36	Fernández, K	The Effect of Different Extraction Techniques on Extraction Yield, Total Phenolic, and Anti-radical Capacity of Extracts from *Pinus radiata* Bark	149
PHYTOCHEMISTRY	36	Matsuhiro, B	Carrageenans From Chilean Samples of *Stenogramme interrupta* (Phyllophoraceae): Structural Analysis and Biological Activity	129
FOODS	35	García, P and Fredes, C	Recovery of Bioactive Compounds from Pomegranate (*Punica granatum* L.) Peel Using Pressurized Liquid Extraction	85
FOOD RESEARCH INTERNATIONAL	32	Pérez-Correa, JR	Phlorotannins: From Isolation and Structural Characterization, to the Evaluation of Their antidiabetic and anticancer potential	64
FRONTIERS IN PHARMACOLOGY	29	Echeverría, J	Chemical Profiling, Antioxidant, Anticholinesterase, and Antiprotozoal Potentials of *Artemisia copa* Phil. (Asteraceae)	33
PHYTOTHERAPY RESEARCH	29	Garrido, G	Analgesic and Anti-Inflammatory Effects Of *Mangifera indica* L. extract (Vimang)	133
FOOD BIOSCIENCE	28	Orellana-Palma, P	Impact of Block Cryoconcentration on Polyphenol Retention in Blueberry Juice	48

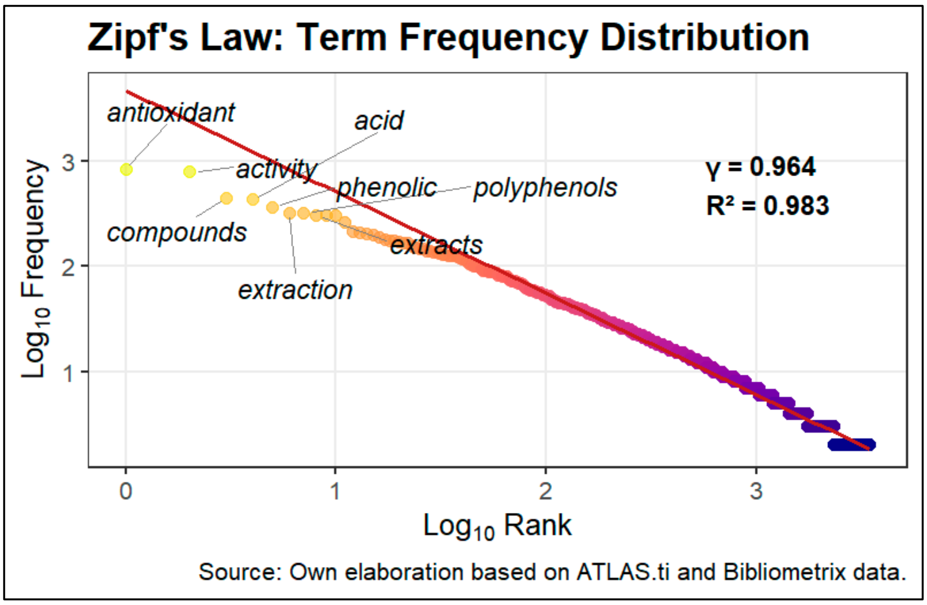

Figure S7. Log–log rank–frequency distribution (Zipf's law) of terms extracted from combined Author Keywords (DE) and Keywords Plus (ID) in the retrieved articles (1976–2025).

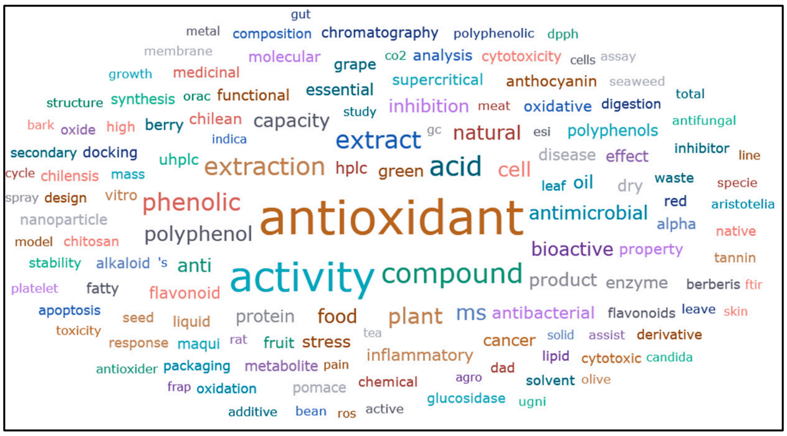

Figure S8. Word cloud showing the most frequent terms extracted from 1371 research titles in the period 2016–2025. The size of the words represents their frequency of occurrence. **Note**: Only terms with a frequency of 10 or more occurrences are displayed to ensure the representation of significant thematic trends.

The word cloud for the 2016–2025 period reveals clear scientific maturity in Chilean phytochemistry. In comparison with titles from the 1970s and 1980s, where basic isolation and natural products chemistry, in its most descriptive dimension, predominated, this latest period is oriented toward evaluating functional bioactivity. With 316 mentions, the term "antioxidant" emerges as the central axis, signaling a shift from descriptive questions like "What compounds does this plant contain?" to more applied inquiries such as "How do these compounds protect cells from oxidative damage?". In this context, methodologies like ORAC (16 mentions), DPPH (14), and FRAP (7) gain prominence, reflecting growing standardization of protocols for evaluating antioxidant capacity and free radical scavenging—features virtually absent in the early decades.

The co-occurrence of terms such as "polyphenol" and "phenolic" (189 combined mentions), alongside "activity" (256 mentions), as well as "polyphenol" (78), "phenolic" (111), "flavonoid" (42), "tannin" (20), and "polyphenolic" (17), suggests a paradigm shift. Phenolic compounds are now studied not only for their chemical structure but also for their biological relevance, including antioxidant, antimicrobial, anti-inflammatory, and antidiabetic activities.

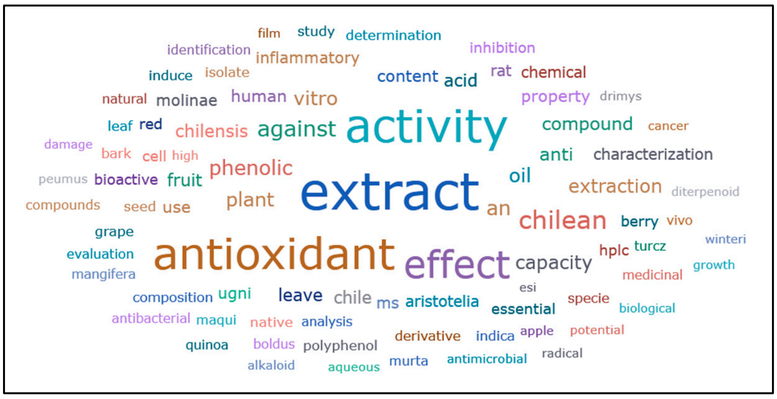

Figure S9. Word cloud showing the most frequent terms extracted from 514 research titles during 2006–2015. The size of the words represents their frequency of occurrence. Note: Only terms with a frequency of 10 or more occurrences are displayed to ensure the representation of significant thematic trends.

The word cloud for the 2016–2025 period shows clear consolidation of Chilean phytochemistry around functional and methodological approaches. The term "extract" (133 mentions) leads by a wide margin, indicating that much of the scientific effort centers on obtaining extracts and evaluating their overall effects. In line with this, "activity" (113), "antioxidant" (107), and "effect" (91) stand out, evidencing emphasis on validating bioactivity—particularly antioxidant capacity. Similarly, the presence of "extraction" (31) and "capacity" (31) reinforces the importance of associated processes and metrics in these assays. Finally, the emergence of "ms" (20) suggests advances in analytical sophistication, incorporating mass spectrometry-coupled techniques for more precise chemical characterization. Overall, this period reflects a transition toward more standardized, quantitative, and technologically advanced research.

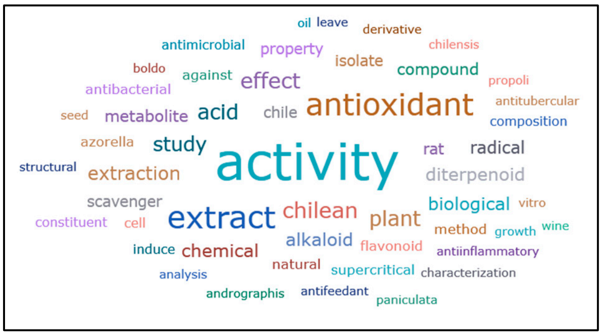

Figure S10. Word cloud showing the most frequent terms extracted from 198 research titles from 1996–2005. The size of the words represents their frequency of occurrence. Note: Only terms with a frequency of 5 or more occurrences are displayed to ensure the representation of significant thematic trends.

From the titles of 198 publications in the 1996–2005 period, considering a total of 2299 terms and applying a minimum threshold of 5 occurrences per word, low-frequency terms were filtered out to highlight the semantically most significant concepts in the scientific output.

The results show that the term "activity" (46 mentions) emerges as the dominant conceptual core, confirming a clear orientation toward the evaluation of bioactivity in phytochemical research. This finding aligns with the significant presence of terms such as "antioxidant" (24), "extract" (25), and "plant" (14), which structure a thematic axis centered on the functional characterization of plant extracts. Collectively, these terms suggest an approach in which plants are viewed not only as sources of compounds but also as complex matrices evaluated for their biological effects.

Similarly, the frequency of terms such as "acid" (14), "compound" (9), "alkaloid" (11), and "diterpenoid" (11) indicates that while the functional approach predominates, chemical characterization remains relevant. However, this characterization appears subordinated to the evaluation of biological properties, reinforcing the transition from descriptive chemistry to applied chemistry.

In parallel, the presence of methodological terms such as "extraction," "method," "analysis," and "characterization" reflects the consolidation of standardized experimental approaches. These terms, along with others such as "in vitro," "radical," and "scavenger," demonstrate the systematic use of biochemical assays to measure specific activities, particularly antioxidant activities.

Another relevant aspect is the emergence of terms associated with specific biological activities, such as "antimicrobial," "antiinflammatory," and "antibacterial," suggesting functional diversification in research lines. This indicates that while antioxidant activity remains central, there is an expansion into other biomedical applications.

Finally, the inclusion of geographical terms such as "chilean" and taxonomic terms such as "boldo" or "azorella" reflects a territorial identity in the research, highlighting the use of native species and interest in valorizing local biodiversity.

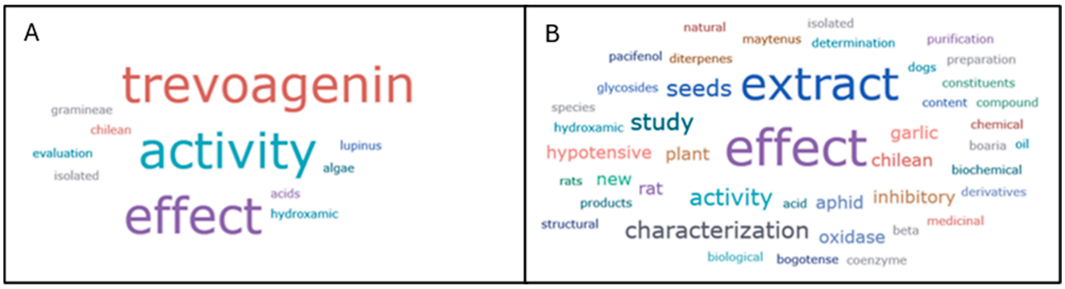

Figure S11. Word cloud showing the most frequent terms extracted from research titles during the early development of Chilean phytochemistry (1976–1995). (A) Word cloud showing the most frequent terms extracted from research titles published during 1976–1985. (B) Word cloud showing the most frequent terms extracted from research titles published during 1986–1995. The size of each word is proportional to its frequency of occurrence. Note: Only terms with a minimum frequency of two occurrences are displayed to highlight the most representative thematic trends.

Figure S11A (1976–1985). The word cloud, built from 16 articles comprising 144 title terms, reflects the foundational stage of Chilean phytochemistry, characterized by a strong emphasis on the identification and characterization of individual natural compounds. The predominance of trevoagenin indicates that research was largely organized around specific molecules rather than broad metabolite classes. Terms such as activity and effect suggest an early interest in evaluating

biological properties, although within a limited and relatively specialized scope. The occurrence of hydroxamic acids, acids, isolated, and evaluation highlights the predominance of classical natural products chemistry focused on compound isolation and structural characterization. Meanwhile, terms such as Lupinus, algae, Gramineae, and Chilean illustrate the initial exploration of native biodiversity as a source of bioactive compounds. Overall, this period represents the methodological foundation of Chilean phytochemistry, centered on compound discovery and preliminary biological evaluation.

Figure S11B (1986–1995). The word cloud, constructed from 50 articles and 508 title terms, illustrates an intermediate stage in the evolution of Chilean phytochemistry, marked by a gradual transition from descriptive natural products chemistry toward research increasingly focused on biological activity. The most frequent terms, effect and extract (8 occurrences each), indicate that studies began to integrate extract preparation with the evaluation of their biological effects. The presence of characterization and activity (4 occurrences each) further reflects the growing linkage between chemical identification and functional assessment. Terms such as hypotensive and inhibitory (3 occurrences each) reveal the emergence of pharmacological research targeting specific biological mechanisms. Additional words, including plant, seeds, compound, chemical, biochemical, and derivatives, demonstrate that plant chemistry remained central, while terms such as rats, aphids, and dogs indicate the increasing incorporation of experimental biological models. Collectively, this period represents a methodological consolidation in which extraction, characterization, and bioactivity assessment became progressively integrated, establishing the foundation for the broader functional and pharmacological research that expanded in subsequent decades.

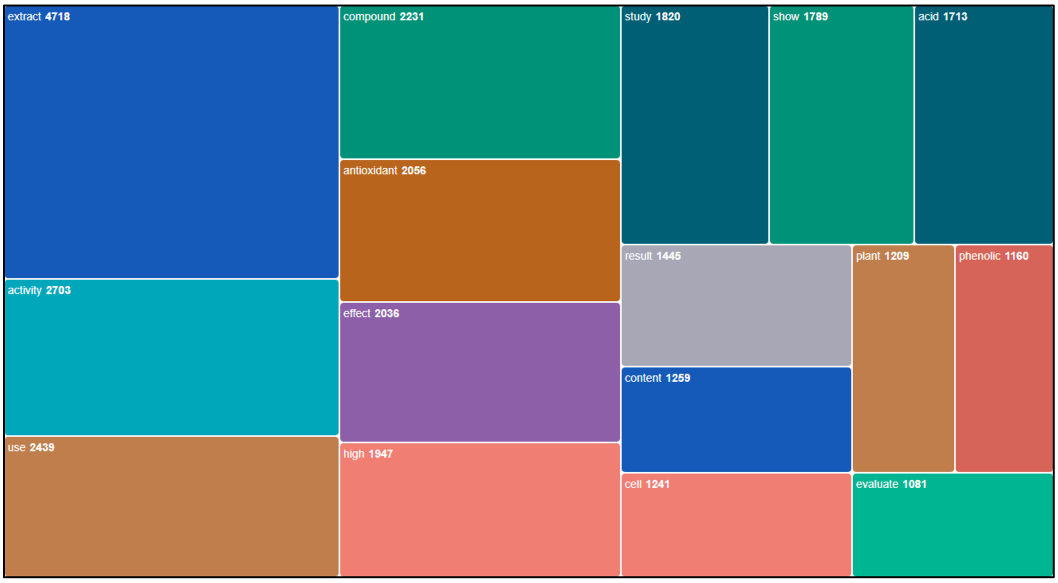

Figure S12. Treemap of terminological frequencies in Chilean phytochemistry literature (1976–2025). This visualization processes a corpus of 439.214 words. Note: Only terms with a frequency of 1000 or more occurrences are displayed to ensure the representation of significant thematic trends.

Structural analysis of the dominant terms (>1,000 mentions)

Frequency analysis reveals a well-defined thematic hierarchy, in which 16 terms concentrate the conceptual core of nearly five decades of phytochemical research in Chile. These terms can be grouped into three main analytical pillars:

1. **Functional core of applied research**

The three most frequent terms—extract (4,718), activity (2,703), and use (2,439)—define the predominant conceptual sequence in the corpus. Their high recurrence suggests that Chilean phytochemistry is organized primarily around a functional model: the extraction of plant materials, the evaluation of biological activity, and the translation of findings into practical applications.

2. **Chemical-biological axis**

Six terms reflect the analytical priorities of the field and show a strong thematic convergence:

Compound and structure: compound (2,231) and acid (1,713) highlight the emphasis on isolation, characterization, and molecular identification.

Biological functionality: antioxidant (2,056) emerges as the predominant biological property, followed by phenolic (1,160), confirming the central role of phenolic compounds in the corpus.

Experimental validation: effect (2,036) and result (1,445) reflect a sustained orientation toward empirical verification and the measurement of biological responses.

3. **Methodological and operational framework**

The remaining terms describe the experimental and methodological context of the studies:

Dominant scientific processes: study (1,820), show (1,789), content (1,259), and evaluate (1,081) represent recurring actions associated with experimental design and quantitative analysis.

Biological object and validation system: plant (1,209) and cell (1,241) indicate that the research begins with plant matrices and progresses toward validation in cellular models, reflecting a shift toward more complex biological approaches.

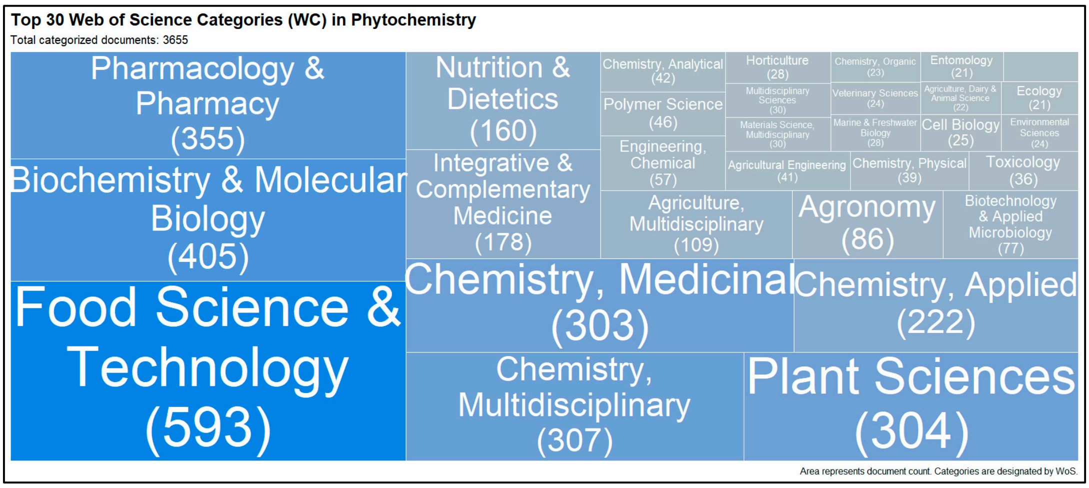

Figure S13. Illustrates the distribution of the 30 most frequent Web of Science categories (WC) among Chilean phytochemistry publications from 1976–2025, using a treemap where rectangle size reflects document counts.

The Web of Science category distribution shows a highly interdisciplinary structure in Chilean phytochemistry, led by *Food Science & Technology* (593 publications), followed by *Biochemistry & Molecular Biology* (405), *Pharmacology & Pharmacy* (355), *Chemistry, Multidisciplinary* (307), *Plant Sciences* (304), and *Chemistry, Medicinal* (303). This pattern indicates that the field rests on a convergence of food science, molecular biosciences, pharmacology, and applied chemistry, reflecting

a predominant focus on bioactive compounds with applications in health, nutrition, and the valorization of plant resources.

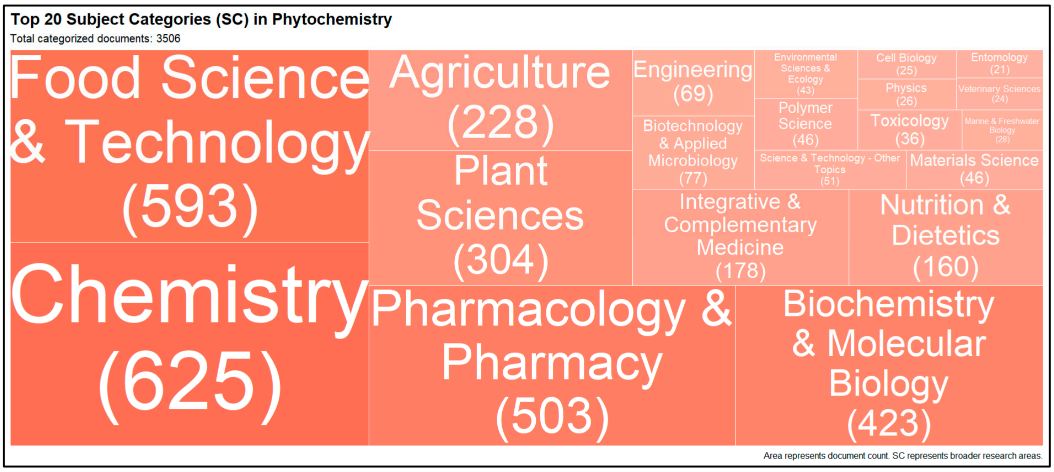

Figure S14. Shows the distribution of the 20 most frequent research areas (SC) in Chilean phytochemistry literature (1976–2025), using a treemap where rectangle size corresponds to publication frequency from Web of Science broader subject classifications.

The distribution of the Subject Categories confirms the multidisciplinary nature of Chilean phytochemistry, with *Chemistry* (625 publications) and *Food Science & Technology* (593) leading, followed by *Pharmacology & Pharmacy* (503), *Biochemistry & Molecular Biology* (423), and *Plant Sciences* (304). These areas concentrate the field's thematic core and show that the research is structured primarily around the chemistry of natural compounds, their biological characterization, pharmacological applications, and use in functional foods, while also integrating agricultural and biomolecular dimensions that reinforce its applied orientation.

Table S5. Indicators of technological and translational maturity in the Chilean phytopharmaceutical sector.

**Dimension**	**Indicator**	**Implications for technological maturity**
Clinical translation	Fewer than 10 clinical studies with Chilean affiliation, mostly focused on dietary supplementation	Limited clinical validation of phytotherapeutic products
Intellectual property	Fewer than 20 related patents identified	Low level of technological appropriation and innovation
Phytopharmaceutical development	112 registered phytopharmaceutical products, only 3 based on native species (*Peumus boldus* Mol., *Buddleja globosa* Hope, and *Haplopappus* spp.)	Limited conversion of native biodiversity into regulated products
Public healthcare integration	Limited availability of scientifically validated phytopharmaceuticals, particularly those based on native Chilean species	Weak integration of phytotherapy into the public healthcare system and limited access for healthcare professionals

Regulatory incorporation	Only 14.4% of Traditional Herbal Medicines (THM) species are endemic, and 28.8% are native; only 8 native species are included in the Chilean Pharmacopoeia	Underrepresentation of native medicinal flora in the formal regulatory framework
Industrial ecosystem	Approximately 15 national companies, predominantly small and medium-sized enterprises	Limited industrial scale-up and innovation capacity
Commercial translation	Exports are dominated by low-value-added products, such as boldo leaves and *Quillaj*a bark and crude extracts	Limited development of high-value-added phytopharmaceutical and biotechnological products

Table S6. Regulatory status, principal bioactive compounds, current applications, and future research opportunities of *Peumus boldus* Mol.

**Category**	**Information**
**Species**	*Peumus boldus* Mol.
**Regulatory status**	Yes
**Traditional use**	Yes
**Market scope**	International
**Main bioactive compounds**	Boldine, laurolitsine, *N*-methyllaurotetanine, isocorydine, laurotetanine, coclaurine, catechins, epicatechin, procyanidin B1, procyanidin B2, procyanidin C1, flavonoids, and essential oil constituents including 1,8-cineole, *p*-cymene, and ascaridole.
**Current applications and uses**	Hepatoprotective, antioxidant, anti-inflammatory, and cytoprotective.
**Research gaps and future opportunities**	Clinical validation; pharmacokinetic and toxicological characterization; development of standardized extracts; sustainable production systems; medicinal biomass cultivation to reduce pressure on natural populations; improved valorization of terpenes, polyphenols and alkaloids throughout the production chain; elucidation of biosynthetic pathways; and integration of computational approaches and artificial intelligence for metabolite discovery and optimization.

Table S7. Regulatory status, principal bioactive compounds, current applications, and future research opportunities of *Quillaja saponaria* Mol.

**Category**	**Information**
**Species**	*Quillaja saponaria* Mol.
**Regulatory status**	Yes
**Traditional use**	Yes
**Market scope**	International
**Main bioactive compounds**	Triterpenoid saponins (including QS-7, QS-17, QS-18, QS-21 and related quillaic acid glycosides), piscidic acid, rutin, other polyphenols, and phenolic acids.
**Current applications and uses**	Vaccine adjuvants, biotechnology, food, and industrial applications.
**Research gaps and future opportunities**	Sustainable production systems; eco-physiological responses to silvicultural management; metabolic plasticity; conservation of genetic diversity; optimization of QS-21 and quillaic acid production; metabolomic traceability; microbiome interactions; climate-change adaptation; optimization of saponin extraction and purification; diversification of high-value applications; development of novel biotechnological products; and integration of multi-omics approaches linking genotype, environment, metabolism, and product quality.

Table S8. Regulatory status, principal bioactive compounds, current applications, and future research opportunities of *Buddleja globosa* Hope.

**Category**	**Information**
**Species**	*Buddleja globosa* Hope
**Regulatory status**	Yes
**Traditional use**	Yes
**Market scope**	National

**Main bioactive compounds**	Verbascoside, linarin, aucubin, catalpol, and other phenylpropanoid glycosides.
**Current applications and uses**	Wound healing, anti-inflammatory, and traditional medicine.
**Research gaps and future opportunities**	Clinical validation of wound-healing, anti-inflammatory, and antimicrobial properties; extract standardization; medicinal cultivation studies; evaluation of environmental influences on chemical composition; pharmaceutical formulation development; and translational studies supporting evidence-based therapeutic applications.

Table S9. Regulatory status, principal bioactive compounds, current applications, and future research opportunities of *Aristotelia chilensis* (Mol.) Stuntz.

**Category**	**Information**
**Species**	*Aristotelia chilensis* (Mol.) Stuntz
**Regulatory status**	Yes
**Traditional use**	Yes
**Market scope**	International
**Main bioactive compounds**	Anthocyanins (delphinidins and cyanidins), alkaloids, flavonoids, and phenolic acids.
**Current applications and uses**	Antioxidant, metabolic health, nutraceuticals, and dietary supplements.
**Research gaps and future opportunities**	Clinical validation of isolated bioactive compounds; further characterization of alkaloids; standardization of leaf-derived products; pharmacokinetic evaluation; integration of computational approaches for bioactive compound assessment; development of medicinal cultivation systems focused on secondary metabolite production; and expansion of functional food and nutraceutical applications.

Table S10. Regulatory status, principal bioactive compounds, current applications, and future research opportunities of *Cryptocarya alba* (Mol.) Looser.

**Category**	**Information**
**Species**	*Cryptocarya alba* (Mol.) Looser
**Regulatory status**	No
**Traditional use**	Yes
**Market scope**	National
**Main bioactive compounds**	Reticuline, catechins (including epicatechin), procyanidins, polyphenols, and aporphine alkaloids.
**Current applications and uses**	Traditional hepatoprotective uses and antioxidant activity.
**Research gaps and future opportunities**	Pharmacological and toxicological validation; chemical standardization; metabolomic characterization; development of sustainable cultivation systems; and advancement of evidence-based phytopharmaceutical and nutraceutical applications.

Table S11. Regulatory status, principal bioactive compounds, current applications, and future research opportunities of *Drimys winteri* J.R.Forst. & G.Forst.

**Category**	**Information**
**Species**	*Drimys winteri* J.R.Forst. & G.Forst.
**Regulatory status**	Yes
**Traditional use**	Yes
**Market scope**	National
**Main bioactive compounds**	Drimane-type sesquiterpenes, essential oils, and flavonoids.
**Current applications and uses**	Antimicrobial, anti-inflammatory, and traditional medicine.

**Research gaps and future opportunities**	Toxicological and safety assessment; chemical standardization; identification of bioactive markers; pharmacological validation; medicinal cultivation studies; evaluation of environmental influences on chemical composition; preclinical and clinical research; integration of computational approaches for bioactive compound evaluation; and translational studies supporting evidence-based therapeutic applications.

Table S12. Regulatory status, principal bioactive compounds, current applications, and future research opportunities of *Laurelia sempervirens* (Ruiz & Pav.) Tul.

**Category**	**Information**
**Species**	*Laurelia sempervirens* (Ruiz & Pav.) Tul.
**Regulatory status**	No
**Traditional use**	Yes
**Market scope**	National
**Main bioactive compounds**	Laurotetanine and related alkaloids, lignans, terpenoids, and polyphenolic compounds.
**Current applications and uses**	Antimicrobial, antioxidant, and traditional spiritual uses.
**Research gaps and future opportunities**	Toxicological and safety assessment; pharmacological validation; development of chemical fingerprints; quality standardization; sustainable production systems; medicinal cultivation studies; evaluation of environmental influences on chemical composition; integration of computational approaches for bioactive compound evaluation; and translational studies supporting evidence-based therapeutic applications.

Table S13. Regulatory status, principal bioactive compounds, current applications, and future research opportunities of *Haplopappus* spp. (Bailahuén).

**Category**	**Information**
**Species**	*Haplopappus* spp. (Bailahuén)
**Regulatory status**	Yes

**Traditional use**	Yes
**Market scope**	National
**Main bioactive compounds**	Flavonoids, diterpenes, coumarins (including prenyletin), and phenolic compounds.
**Current applications and uses**	Digestive, hepatoprotective, and anti-inflammatory uses.
**Research gaps and future opportunities**	Advanced chemical characterization; chemical standardization; identification of bioactive markers; pharmacological and clinical validation; development of medicinal cultivation systems; and production of standardized phytotherapeutic products.

Table S14. Regulatory status, principal bioactive compounds, current applications, and future research opportunities of *Kageneckia oblonga* Ruiz & Pav.

**Category**	**Information**
**Species**	*Kageneckia oblonga* Ruiz & Pav.
**Regulatory status**	No
**Traditional use**	Yes
**Market scope**	National
**Main bioactive compounds**	Flavonoids, pomolic acid, ursolic acid, prunasin, and cucurbitacins.
**Current applications and uses**	Traditional treatment of fevers, renal and hepatic disorders, and digestive ailments.
**Research gaps and future opportunities**	Toxicological and safety assessment; pharmacological validation; development of chemical fingerprints; quality standardization; sustainable production systems; medicinal cultivation studies; characterization of chemical variability and environmental influences on metabolite composition; integration of computational approaches for bioactive compound evaluation; and translational studies supporting evidence-based therapeutic applications.

Table S15. Regulatory status, principal bioactive compounds, current applications, and future research opportunities of *Schinus latifolius* (Gillies ex Lindl.) Engl.

**Category**	**Information**
**Species**	*Schinus latifolius* (Gillies ex Lindl.) Engl.
**Regulatory status**	No
**Traditional use**	Yes
**Market scope**	National
**Main bioactive compounds**	Flavonoids (including sophoricoside).
**Current applications and uses**	Traditional use for relieving stomach pain.
**Research gaps and future opportunities**	Toxicological and safety assessment; pharmacological validation; comprehensive phytochemical characterization of the whole plant; development of chemical fingerprints; quality standardization; medicinal cultivation studies; evaluation of chemical variability and environmental influences on metabolite composition; integration of computational approaches and network pharmacology for bioactive compound evaluation; sustainable production systems; and translational studies supporting evidence-based therapeutic applications.

Note: Regulatory status refers to the species' inclusion in the current Chilean Traditional Herbal Medicines regulatory framework. Although each species presents specific research opportunities, common challenges across most native Chilean medicinal plants include limited pharmacological and toxicological evidence, insufficient chemical standardization and quality control, scarce pharmacokinetic and clinical studies, the need for sustainable production systems, and the incorporation of emerging technologies such as metabolomics, computational approaches, and artificial intelligence to accelerate phytochemical discovery and phytopharmaceutical development.

Table S16. *Peumus boldus* publications omitted from the topic-based search.

**Title**	**Year**
Peumus boldus Used in the Synthesis of ZnO Semiconductor Nanoparticles and Their Evaluation in Organic Contaminants	2023
Direct identification of phenolic constituents in Boldo Folium (*Peumus boldus* Mol.) infusions by high-performance liquid chromatography with diode array detection and electrospray ionization tandem mass spectrometry	2010
Herbicidal Activity of *Peumus boldus* and *Drimys winterii* Essential Oils from Chile	2011

Effect of boldo (*Peumus boldus* Molina) infusion on lipoperoxidation induced by cisplatin in mice liver	2009
Composition and antimicrobial activity of the essential oil of *Peumus boldus* leaves	1999
Chemical profile and antimicrobial activity of boldo (Peumus boldus Molina) extracts obtained by compressed carbon dioxide extraction	2008
Effect of vacuum storage on shelf life of a grain protector based on Peumus boldus Molina foliage powder and lime against Sitophilus zeamais Motschulsky	2014
Insecticidal properties of *Peumus boldus* Molina powder used alone and mixed with lime against *Sitophilus zeamais* Motschulsky (coleoptera: curculionidae)	2009
Boldo restores vascularization and reduces skeletal muscle inflammation in symptomatic mice with dysferlinopathy	2025
Addition of *Trichoderma* consortia to Chilean endemic flora compost teas strongly enhances *in vitro* and *in vivo* biocontrol of phytopathogenic fungi	2023
Antiproliferative effect of boldine on neural progenitor cells and on glioblastoma cells	2023
Boldine Attenuates Synaptic Failure and Mitochondrial Deregulation in Cellular Models of Alzheimer's Disease	2021
Telomerase Inhibition by a New Synthetic Derivative of the Aporphine Alkaloid Boldine	2018
Antiinflammatory and antipyretic effects of boldine	1994
Antioxidant screening of medicinal herbal teas	2006
A novel alkaloid antioxidant, Boldine and synthetic antioxidant, reduced form of RU486, inhibit the oxidation of LDL *in-vitro* and atherosclerosis *in vivo* in LDLR-/- mice	2004
Boldine Improves Kidney Damage in the Goldblatt 2K1C Model Avoiding the Increase in TGF-β	2018
Evaluation of Chilean Boldo Essential Oil as a Natural Insecticide Against *Chrysomya megacephala* (Diptera: Calliphoridae)	2020
Protective effect of boldine in experimental colitis	1997
Activity of boldine on rat ileum	1991
